# Jumping into recovery: A systematic review and meta‐analysis of discriminatory and responsive force plate parameters in individuals following anterior cruciate ligament reconstruction during countermovement and drop jumps

**DOI:** 10.1002/jeo2.12018

**Published:** 2024-04-02

**Authors:** Wasim Labban, Thaer Manaseer, Eric Golberg, Mark Sommerfeldt, Stephanie Nathanail, Liz Dennett, Lindsey Westover, Lauren Beaupre

**Affiliations:** ^1^ Department of Physiotherapy, Faculty of Rehabilitation Medicine University of Alberta Edmonton Canada; ^2^ Mirdif Center for Physiotherapy and Rehabilitation Dubai United Arab Emirate; ^3^ Department of Sport Rehabilitation, Faculty of Physical Education & Sports Sciences The Hashemite University Zarqa Jordan; ^4^ Department of Surgery, Division of Orthopedic Surgery, Faculty of Medicine & Dentistry University of Alberta Edmonton Canada; ^5^ Glen Sather Sports Medicine Clinic University of Alberta Edmonton Canada; ^6^ Orthopedic Surgery Alberta Health Services Edmonton Canada; ^7^ Geoffrey and Robyn Sperber Health Sciences Library University of Alberta Edmonton Canada; ^8^ Faculty of Engineering University of Alberta Edmonton Canada

**Keywords:** ACLR, anterior cruciate ligament, anterior cruciate ligament reconstruction, countermovement jump, drop jump, force plate, ground reaction force, jump height, kinetics

## Abstract

**Purpose:**

Comprehensive understanding of force plate parameters distinguishing individuals postprimary anterior cruciate ligament reconstruction (ACLR) from healthy controls during countermovement jumps (CMJ) and/or drop jumps (DJ) is lacking. This review addresses this gap by identifying discriminative force plate parameters and examining changes over time in individuals post‐ACLR during CMJ and/or DJ.

**Methods:**

We conducted a systematic review and meta analyses following the Preferred Reporting Items for Systematic Review and Meta‐Analyses (PRISMA) guidelines. Nine databases were searched from inception to March 2022. We included cross‐sectional papers comparing post‐ACLR with healthy controls or longitudinal studies of individuals at least 6 months postprimary ACLR while performing CMJ and/or DJ on force plates. The methodological quality was appraised using the Modified Downs and Black Checklist.

**Results:**

Thirty‐three studies including 1185 (50.38%) participants post‐ACLR, and 1167 (49.62%) healthy controls, were included. Data were categorised into single‐leg CMJ, double‐leg CMJ, single‐leg DJ, and double‐leg DJ. Jump height was reduced in both single (mean difference [MD] = −3.13; *p* < 0.01; 95% confidence interval [CI]: [−4.12, −2.15]) and double‐leg (MD = −4.24; *p* < 0.01; 95% CI: [−5.14, −3.34]) CMJs amongst individuals with ACLR. Similarly, concentric impulse and eccentric/concentric impulse asymmetry could distinguish between ACLR (MD = 3.42; *p* < 0.01; 95% CI: [2.19, 4.64]) and non‐ACLR (MD = 5.82; *p* < 0.01; 95% CI: [4.80, 6.80]) individuals. In double‐leg DJs, peak vertical ground reaction forces were lower in the involved side (MD = −0.10; *p* = 0.03; 95% CI: [−0.18, −0.01]) but higher in the uninvolved side (MD = 0.15; *p* < 0.01; 95% CI: [0.10, 0.20]) when compared to controls and demonstrated significant changes between 6 months and 3 years post‐ACLR.

**Conclusion:**

This study identified discriminative kinetic parameters when comparing individuals with and without ACLR and also monitored neuromuscular function post‐ACLR. Due to heterogeneity, a combination of parameters may be required to better identify functional deficits post‐ACLR.

**Level of Evidence:**

Level III.

**Abbreviations**
ACL: anterior cruciate ligament
ACLR: anterior cruciate ligament reconstruction
CI: confidence interval
CMJ: countermovement jump
CoP: centre of pressure
DB: Downs and Black
DJ: drop jump
MD: mean difference
OCEBM: Oxford Centre of Evidence‐Based Medicine
PRISMA: Preferred Reporting Items for SystematicReview and Meta‐Analyses
RSI: reactive strength index
RSR: reactive strength ratio
RTS: return to sport
SD: standard deviation
TTS: time to stabilisation
vGRF: vertical ground reaction force

## INTRODUCTION

Anterior cruciate ligament (ACL) rupture is a devastating injury that frequently occurs in sports [14] and accounts for at least 50% of all knee injuries [14, 58]. ACL reconstruction (ACLR) is often recommended to restore joint stability and minimise potential damage to articular cartilage and menisci [2]. The reported proportion of individuals who return to a competitive level of sport following ACLR is 55%, while 81% return to any level of sport [3]. Up to 38% of elite athletes reduce their participation levels or stop their career within 3 years after ACLR [67]. Moreover, 20%–25% of post‐ACLR individuals experience a rerupture or a contralateral ACL injury early during the return to sport (RTS) period [68]. This may be related in part to the lack of standardised, validated RTS criteria to adequately assess RTS capacity.

Kinetic measurement systems, such as force plates, have emerged as popular tools to measure various parameters objectively while performing different movement tasks. These systems use force sensors to quantify forces exerted during activities or tasks [10]. Clinicians and researchers utilise these systems to assess functional progression throughout rehabilitation and to assist in determining the ability to RTS in post‐ACLR individuals [24]. Previous studies have examined various kinetic parameters in the ACLR population. Our previous work identified several parameters assessing different movement tasks such as jumping and landing [4], standing balance [1, 16], gait [62] and other functional tasks [60]. Notably, jumping and landing were the most frequently studied activities in individuals following primary ACLRs [44, 54].

Countermovement jumps (CMJ) and drop jumps (DJ) have been widely used in the literature to assess performance in individuals with ACLR [44]. The CMJ involves a downward movement to a semisquat depth position before extending the back, hips and knees to jump vertically as high as possible. The DJ involves dropping down from a box, followed immediately by jumping vertically as high as possible. Several studies utilised those jumps to identify risk factors associated with sports injuries [54], assess association with other measures of performance [5], detect neuromuscular fatigue [5] and to quantify the functional consequences during sports rehabilitation [5], particularly, following ACLR.

Assuming that ACLR causes neuromuscular impairments that can be detected with a force plate while performing CMJ and/or DJ [45], it is essential to understand which force plate parameters can best detect any functional impairments or deficits. Therefore, the primary objective of the current systematic review was to identify force plate parameters that are discriminative between individuals following primary ACLR and healthy controls while performing CMJ and DJs. The secondary objective was to identify force plate parameters that are responsive to changes in neuromuscular function over time in individuals following primary ACLR while performing CMJs and DJs. Based on existing literature, it is hypothesised that kinetic force plate parameters are significantly different between individuals following ACLR and healthy controls during CMJ and DJ. Additionally, it is hypothesised that these force plate parameters could demonstrate responsiveness to changes in neuromuscular function over time in individuals following primary ACLR. Findings from the current systematic review may provide clinicians and researchers with objective outcomes to inform RTS decisions in individuals following ACLR.

## METHODS

### Registration

This systematic review was registered on the Open Science Framework https://doi.org/10.17605/OSF.IO/7FTQP.

### Framework

The authors conducted and reported the current systematic review according to the Preferred Reporting Items for Systematic Review and Meta‐Analyses (PRISMA) guidelines [52] and PRISMA‐Search extension [57].

### Eligibility criteria

All inclusion and exclusion criteria are reported in Table 1. The constructs of ‘participants’, ‘primary ACLR’ and ‘kinetic measurement systems’ are operationalised in Table 2.

**Table 1:** Inclusion and exclusion criteria.

Inclusion criteria	Exclusion criteria
Human participants	Animal model, cadaver, simulated or computer models
Original or primary quantitative data (cross‐sectional with a healthy control group, longitudinal with at least one kinetic force‐plate measurement at a minimum of two different time points)	Not primary data (e.g., systematic review, literature review, meta‐analysis, editorial, commentary, opinion papers or conference proceedings)
Primary ACLR—With the measurement taken at least, 6 months post‐ACLR	Case report
At least one kinetic parameter measured solely by a force plate	Cross‐sectional study with no control group. Exclude if the control is the contralateral limb
Performed drop jump or countermovement jump	Secondary ACLR (in ipsilateral or contralateral knee)
	Concomitant significant injuries or surgical interventions to the medial or lateral collateral ligament
	Skeletally immature participants
	Congenital deformities
	Other musculoskeletal problems that could influence the force plate parameters including foot disorders, hip disorders and lower back and pelvic problems
	Neurological problems that could affect balance or neuromuscular co‐ordinations
	ACL repair (not reconstruction) where the ACL was reattached.
	Parameters measured with tools that do not employ force plates technology (motion capture system, isokinetic systems, infrared contact mats)
	Kinetic parameters that cannot be measured with force plates solely (joint moments)
	Other types of jumps or other functional activities such as walking, running, squatting, cutting, pivoting and so on

Abbreviations: ACL, anterior cruciate ligament; ACLR, anterior cruciate ligament reconstruction.

**Table 2:** Definitions.

Participants	ACLR group: Any individual who reached skeletal maturity with at least 6 months history of primary ACLR; no limitation to age, sex, sport played or activity level. Healthy control group: healthy uninjured individuals who reached skeletal maturity with no ACLR history; no limitation to age, sex, sport played or activity level.
Primary ACLR	A first‐time ACLR; surgical tissue graft replacement of the anterior cruciate ligament to restore its function after injury [43].
Drop jump	Jumping/descending of a box placed behind a force plate, land on the force plate and jump vertically for a maximum height [32].
Countermovement jump	From standing position, participant performs a downward motion to specific/self‐selected depth before reversing the motion by triple extending the hip, knee and ankle, jumping up for a maximum height [56].

Abbreviations: ACL, anterior cruciate ligament; ACLR, anterior cruciate ligament reconstruction.

### Information sources and search strategy

A research team member (W. L.) and health sciences librarian (L. D.) developed an extensive list of search terms for each construct. The health sciences librarian (L. D.) conducted searches in Medline (Ovid MEDLINE(R) ALL), Embase (Ovid interface), CINAHL Plus with Full Text (EBSCOhost interface), Web of Science Core Collection (Indexes = SCI‐EXPANDED, SSCI, A&HCI, ESCI), SCOPUS, Proquest Dissertations and Theses Full text, Pubmed Central, Science Direct and Google Scholar from database inception until 13 March 2022. The search combined subject headings (where available) and keywords for the concepts of (1) ACL‐R, (2) vertical jumps and (3) movement properties. The movement properties construct was searched in the full text if databases allowed for it. The search strategy was optimised for each database. No language or date limits were applied but conference abstracts were removed. Reference lists of included articles and other relevant reviews were reviewed for additional studies. The full search strategy is available in Supporting Information S1: Appendix 1.

### Selection process

Records were imported into EndNote V.XI. After duplicate removal, records were imported into Covidence platform (Covidence, Veritas Health Innovation). The authors W. L. and T. M. independently screened title and abstracts to determine potential relevant records, followed by full‐text review to determine final record selection. Any disagreement between the two authors was resolved through consensus. Consultation with a third author was not needed. All decision and exclusion reasons were recorded on Covidence.

### Data extraction

The authors W. L. and T. M. performed data extraction independently in duplicate, using a structured data extraction form on (Google Sheets). Discrepancies were resolved through consensus. Data items included study characteristics, sample characteristics, testing protocols and outcomes (see Table 3).

**Table 3:** Data Items.

Category	Item(s)
Study characteristics	Author(s), year of publication, study design, country and language
Participant sample characteristics	Sample size disaggregated by sex, age, reported activity and activity level
Primary ACL surgical details	Graft type, side of surgery (dominant/nondominant), time from surgery
Testing protocol details for each type of jump	Sampling frequency, testing protocol (hand placement, shoes on/off, warm‐up protocol) and number of trials per test
Outcomes with estimates and variances	Parameters that were measured solely by force plates with means and SDs

Abbreviations: ACL, anterior cruciate ligament; SD, standard deviation.

### Quality appraisal

The authors critically appraised the methodological quality of included records using the Modified Downs and Black (DB) checklist [15]. The DB checklist is a quality assessment tool that rates studies based on study design, quality of reporting, internal validity (including potential confounding) and external validity. It employs a 32‐point scoring system (11 points for reporting, three points for external validity, seven points for bias, six points for confounding and five for power [one point for power in the modified version]) [15, 38]. For observational studies, items number 4, 8, 13, 14, 19, 23 and 24 on the checklist (adding up to seven points) are not applicable. The tool was selected for its reported intrarater and inter‐rater reliability [15].

The authors used the Oxford Centre of Evidence‐Based Medicine (OCEBM) 2011 model [22] to identify the level of evidence that the included records represented. The OCEBM 2011 model is simple, and its structure reflects clinical decision making [29]. Discrepancies in DB scoring or OCEBM categorisation were resolved by consensus between the two reviewers (W. L. and T. M.).

### Data synthesis

The extracted data were divided according to the study designs into two main categories: cross‐sectional and longitudinal. Data from longitudinal studies that included control groups were pooled to form cross‐sectional data to reduce the chance of data reporting bias. Similarly, data from cross‐sectional studies comparing between individuals with ACLR and controls at different time points (later than 6 months following ACLR), or male and female individuals with control groups were pooled to form one ACLR group for comparison [59]. The research team estimated the pooled mean and the sum of squares of standard deviation (SD) using the ‘dplyr package’ in R (R v4.1.0, The R Foundation for Statistical Computing).

Then, the authors further subdivided the resulting two study categories into four main groups according to the jump task used including the single‐leg CMJ, double‐leg CMJ, single‐leg DJ and double‐leg DJ with studies assigned accordingly. Data were imported as means and SDs into Review Manager for Meta‐Analysis (RevMan v5.4.1; The Nordic Cochrane Centre). The authors estimated SDs for studies that reported means and 95% confidence intervals (CI) following the Cochrane Handbook for Systematic Reviews of Interventions [25]. The authors used a random effects model with standardised mean differences and 95% CIs. Pooled effect size, 95% CI, *p* value and heterogeneity were calculated per outcome by means of the *I* ^2^ test [26]. *I*² values below 30% indicate mild heterogeneity, values between 30% and 50% suggest moderate heterogeneity and values over 50%, coupled with significant *Q* statistics, imply notable heterogeneity amongst the included studies [26, 27]. We considered sensitivity analysis for meta‐analysis when *I* ^2^ values are greater than 50%. Meta‐analyses were performed for each individual force plate parameter when it was reported with means and SDs in at least two studies.

## RESULTS

### Identification of studies

**Figure 1:**
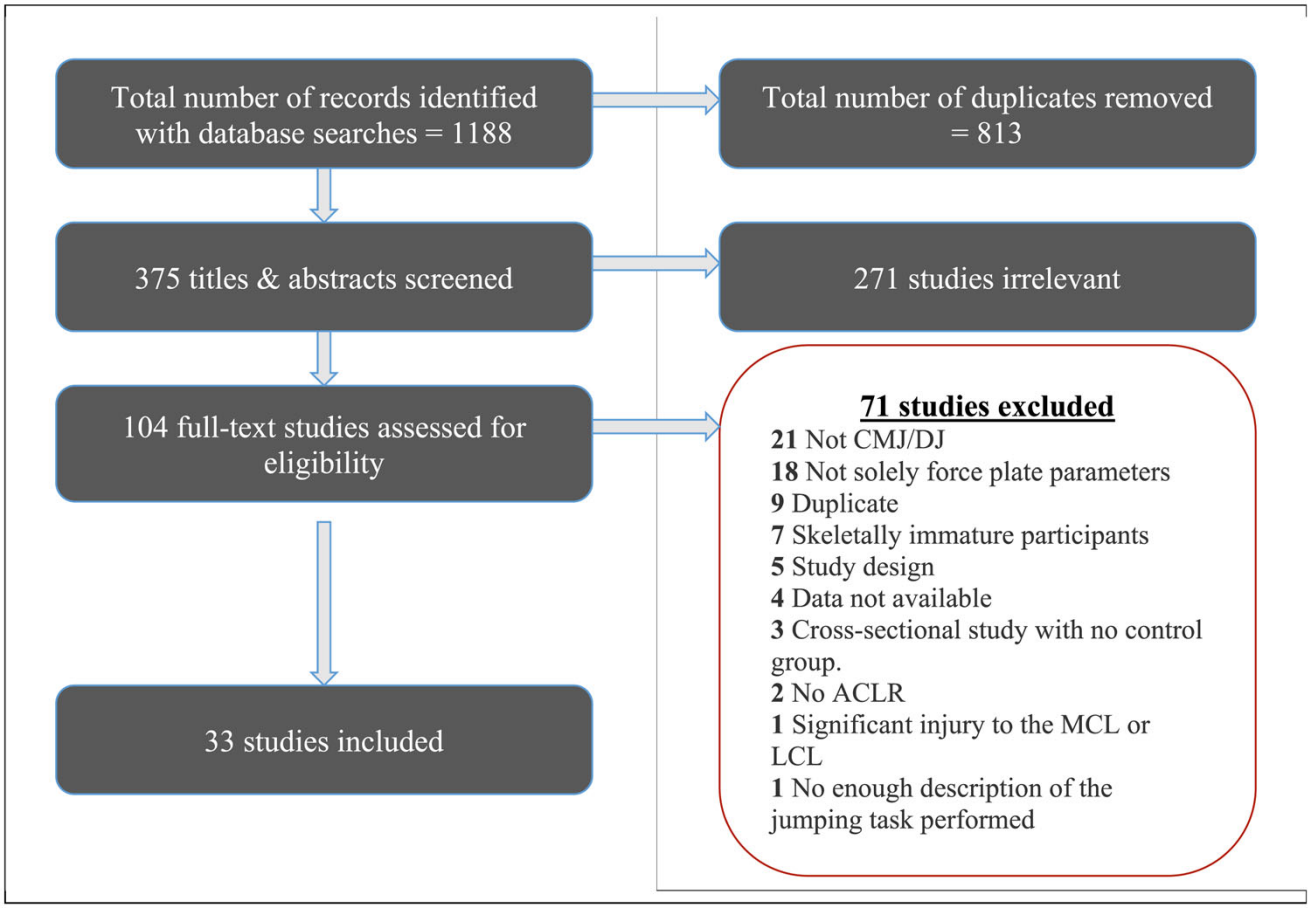
Search results and study selection. ACLR, anterior cruciate ligament reconstruction; CMJ, countermovement jump; DJ, drop jump; LCL, lateral collateral ligament; MCL, medial collateral ligament.

An overview of the study identification process is provided in Figure 1. Of 1188 identified records, 375 unique records underwent title/abstract screening. Of these, 104 were reviewed in full and 33 studies were included. Papers evaluating the same cohort with different (a) aims, (b) tasks evaluated or (c) outcomes were treated independently.

### Studies characteristics

The characteristics of the 33 included studies are summarised in Supporting Information S1: Appendix II. These included 27 cross‐sectional (81.82%) and six longitudinal (18.18%) studies. Studies were published between 2007 and 2022 with 22 (63.64%) studies published since 2018. Studies were conducted in 13 countries with the highest number conducted in the United States (12 [36.64%]). Overall, 2352 participants were included (female = 839 [37.81%]; male = 1380 [62.19%]). The mean age of participants ranged from 16.7 (±3.0) to 31.5 (±7.6) years. Of those, 1185 participants (50.38%) had ACLR, and 1167 (49.62%) were healthy controls, representing a variety of physical activity and sport participation levels. Healthy control groups existed in 29 (87.88%) studies.

Following the methodological considerations recommended in a previous scoping review [44], a lack of reporting and a wide degree of heterogeneity were observed across the included studies in terms of force plate sampling frequency, warming up protocol, shoe‐wearing requirements, hand placement requirements and jumping protocol across the included studies. For instance, the force plate sampling frequencies were reported in 29 (87.9%) studies, and ranged between 500 and 2400 Hz. The most frequently used sampling frequencies were 1000 Hz (*n* = 16 [48.5%]) [12, 13, 28, 37, 39, 40, 41, 46, 47, 50, 51, 55, 56, 63, 64, 65] and 1200 Hz (*n* = 4 [12.1%]) [17, 33, 48, 61]. While varying warm‐up protocols were reported in 17 (51.5%) studies [8, 9, 12, 13, 19, 28, 33, 35, 36, 37, 39, 46, 47, 50, 51, 55, 56], 16 (48.5%) studies did not provide information about a warm‐up protocol. Similarly, participants were instructed to perform the jumping tasks barefoot in two (6.1%) studies [28, 42] and with shoes on in 11 (33.3%) studies [7, 8, 9, 31, 32, 33, 37, 39, 41, 46, 51], while the remaining 20 (60.6%) studies did not specify whether participants wore shoes or not. Related to hand placement during jumping, participants were instructed to keep their hands on hips in 14 (42%) studies [12, 13, 20, 28, 35, 36, 37, 39, 40, 46, 47, 50, 55, 56] or to cross their arms on their chest in one (3%) study [7]. Apart from the variation in the number of trials, single and double‐leg CMJ protocols remained consistent across the studies. However, while performing DJ, four studies reported having their participants jump off a box that was placed at certain distances behind the force plate [19, 31, 32, 41], while the remaining studies instructed their participants to drop off a box onto force plates.

### Quality appraisal and level of evidence

The studies included in the current review showed a maximum evidence level of 4, as per the OCEBM model. This corresponds to cross‐sectional, case‐control or lower‐quality prognostic cohort studies.

In terms of methodological quality, gauged using the DB criteria, the median score was 10/21, with scores ranging from 7/21 to 12/21.

Common methodological limitations amongst the studies included a partial description of primary confounders, potential selection bias (i.e., no clear differentiation between those who chose to participate versus those who did not), small sample sizes and lack of detailed explanation of the validity of the methodological approaches used to evaluate CMJs and DJs.

### Jumping tasks

A total of 38 comparisons were identified, of which 31 compared between individuals with ACLR and healthy controls and seven comparisons studied individuals at different time points at least 6 months post‐ACLR. Means and SDs were used for meta‐analysis (Table 4). One study used the mean differences and degree of freedom, and therefore, was excluded from meta‐analyses, yet its findings were reported narratively. All the parameters utilised to evaluate CMJs and DJs in individuals with and without ACLR are reported in Supporting Information S1: Appendix II. The operational definitions of those parameters are detailed in Supporting Information S1: Appendix III.

**Table 4:** Details of comparisons.

Comparisons	Single‐leg		Double‐leg	Total
Cross‐sectional				
CMJ	4		7	11
DJ	5		15	20^a^
Total cross‐sectional comparisons	9^b^		22	31
Longitudinal				
CMJ	0		1	1
DJ	1		5	6^c^
Total longitudinal comparisons	1		6	7^d^
Total	10		28^e^	38^f^

Abbreviations: CMJ, countermovement jump; DJ, drop jump.

^a^ Twenty comparisons in 19 studies.

^b^ Nine comparisons in eight studies.

^c^ Six comparisons in five studies.

^d^ Seven comparisons in six studies.

^e^ Twenty‐eight comparisons in 26 studies looking at double‐leg DJ.

^f^ Thirty‐eight comparisons in 33 studies.

### Single‐leg CMJ

**Figure 2:**
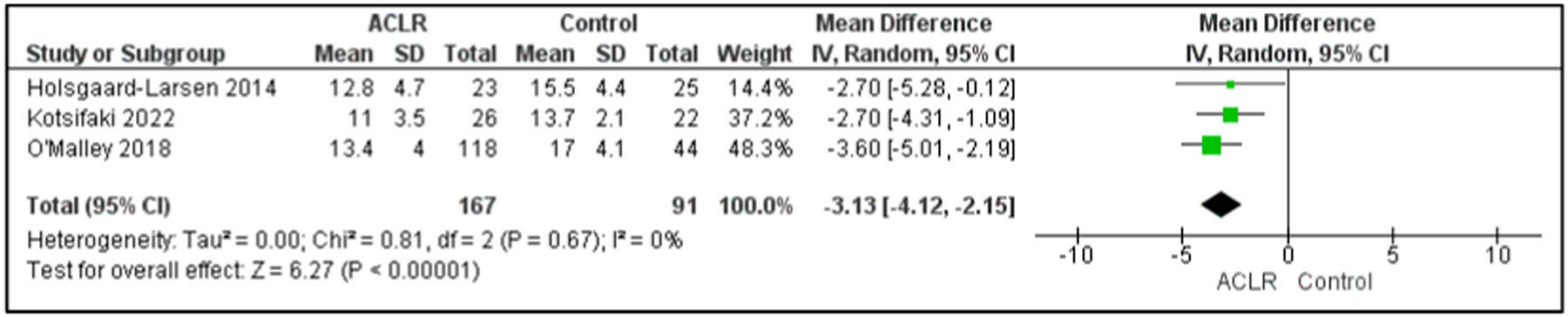
Forest plot comparing jump height while performing single‐leg countermovement jump in individuals with and without anterior cruciate ligament reconstruction (ACLR). CI, confidence interval; SD, standard deviation.

The authors identified four cross‐sectional studies comparing between individuals following ACLR (*n* = 177) with healthy controls (*n* = 108) [20, 28, 39, 50]. All four studies included male participants only. Six parameters were identified (peak vertical ground reaction force (vGRF) [20], centre of pressure length of path [20], time to stabilisation [20], flight time [20], jump height [28, 39, 50] and peak power [50]). Our meta‐analysis, incorporating data from three different studies [28, 39, 50], revealed that jump height was significantly lower in the ACLR compared to the healthy control group with a mean difference (MD) of −3.13 cm (*p* < 0.01; 95% CI: [−4.12, −2.15]) and no observed heterogeneity (*I* ^2^ = 0%) (Figure 2). Peak power was reported in only one comparison demonstrating a lower single‐leg CMJ peak power in the ACLR group compared to the control group [50]. No other parameters demonstrated significant differences between the two groups. There were no longitudinal comparisons of single‐leg CMJ.

### Double‐leg CMJ

**Figure 3:**
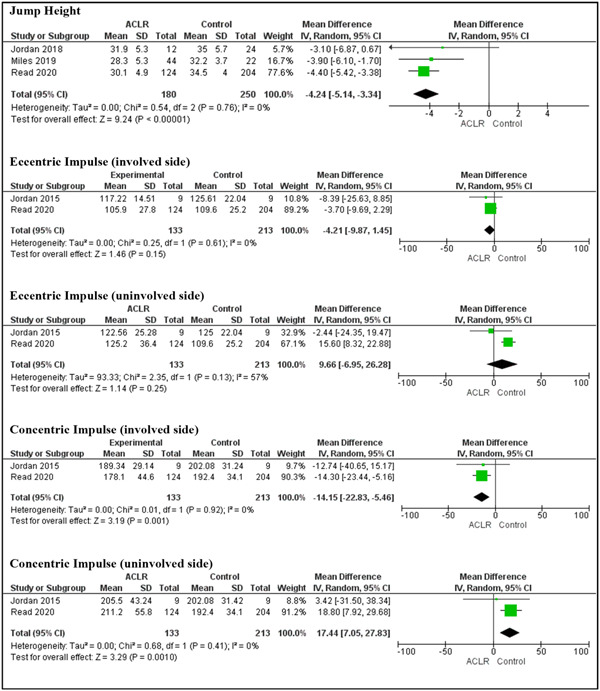
Forest plots comparing jump height, eccentric and concentric impulses while performing double‐leg countermovement jump in individuals with and without anterior cruciate ligament reconstruction (ACLR). CI, confidence interval; SD, standard deviation.

Seven cross‐sectional studies compared between participants post‐ACLR (*n* = 245 [*f* = 35, m = 190) and healthy controls (*n* = 494 [*f* = 214, *m* = 260) [7, 12, 35, 36, 40, 47, 56], with one study not reporting participants' sex [40]. We identified 32 unique parameters across the studies. Jump height was the most commonly reported parameter in four (57.1%) studies [35, 40, 47, 56], followed by impulse concentric [36, 56], impulse concentric asymmetry [40, 56], impulse eccentric [36, 56] and impulse eccentric asymmetry [40, 56] each reported in two (28.6%) studies. One study reported the mean differences instead of means and SDs for jump height and was excluded from the meta‐analysis [40]. The meta‐analysis on the remaining three studies demonstrated lower jump height in the ACLR compared to the healthy control group (MD = −4.24 cm; *p* < 0.01; 95% CI: [−5.14, −3.34]) with no reported heterogeneity (*I* ^2^ = 0%). In two separate studies [36, 56], the involved limb exhibited significantly lower concentric impulse, whereas the uninvolved limb displayed significantly higher concentric impulse compared to the control group, (MD = −14.15 N.s; *p* < 0.01; 95% CI: [−22.84, −5.46]) and (MD = 17.44 N.s; *p* < 0.01; 95% CI: [7.05, 27.83]), respectively (Figure 3).

**Figure 4:**
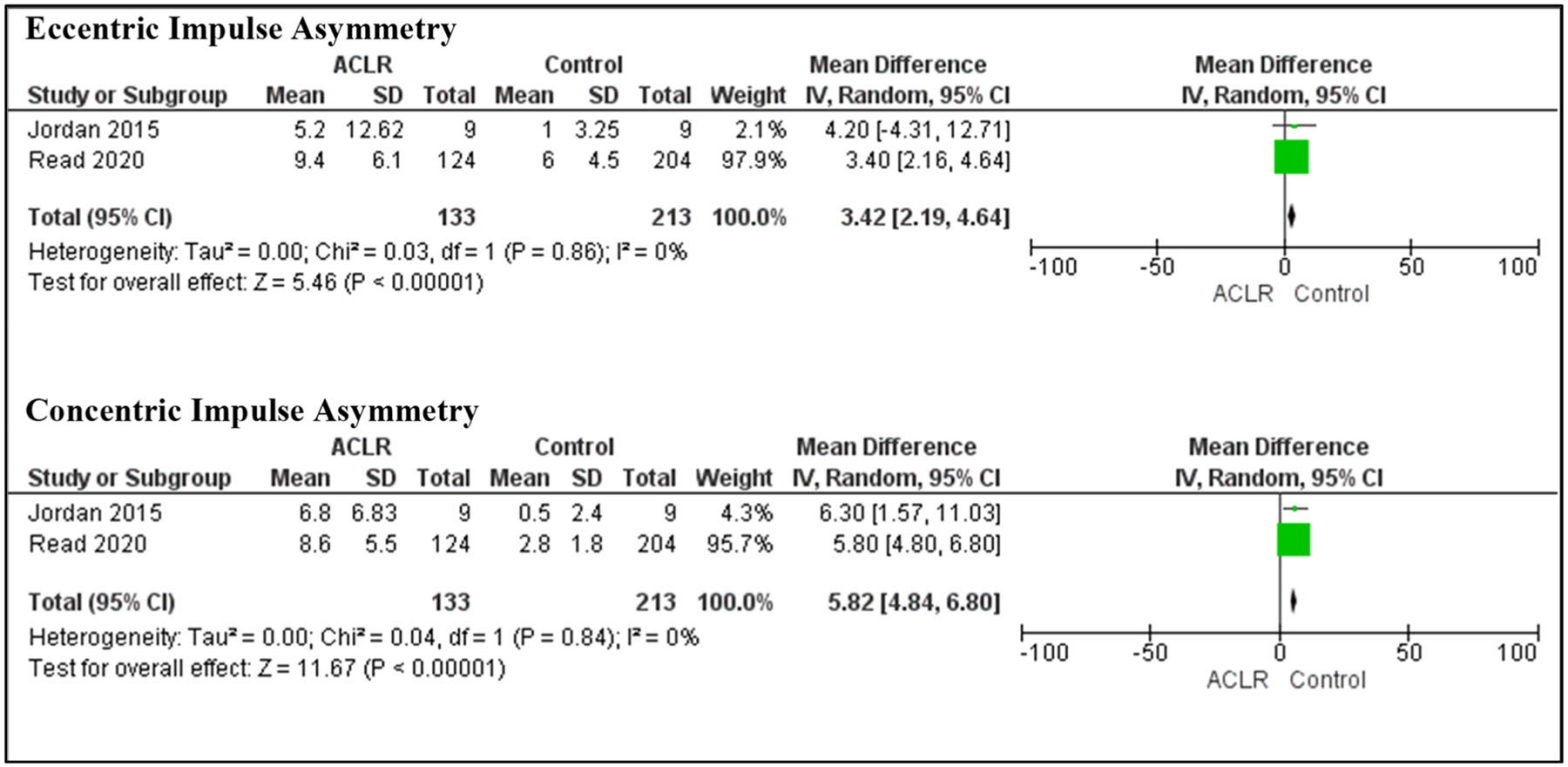
Forest plots comparing eccentric and concentric impulses asymmetry while performing double‐leg countermovement jump in individuals with and without anterior cruciate ligament reconstruction (ACLR). CI, confidence interval; SD, standard deviation.

Eccentric impulse and concentric impulse demonstrated significantly higher asymmetries in the ACLR group compared to the healthy control group, (MD = 3.42; *p* < 0.01; 95% CI: [2.19, 4.64]) and (MD = 5.82; *p* < 0.01; 95% CI: [4.80, 6.80]), respectively (Figure 4). However, when evaluating eccentric impulse in the involved and uninvolved limbs of the ACLR group compared to the control groups, no significant differences were found.

Several parameters were only reported in one study: Concentric impulse_(normalised)_ [47], eccentric impulse_(normalised)_ [47], landing impulse_(normalised)_ [47], concentric peak vGRF [56], eccentric mean GRF [56], eccentric RFD [56], peak power [56], peak vGRF during landing [56], relative peak power [35], velocity at peak power [35], velocity max [35], concentric peak vGRF asymmetry [56], eccentric mean GRF asymmetry [56], eccentric RFD asymmetry [56] and peak vGRF asymmetry during landing [56]. All of these comparisons between ACLR participants and healthy control participants were significantly different favouring the healthy control groups (Supporting Information S1: Appendix II).

One longitudinal study evaluated 44 male participants in a repeated measure design at 6 and 9 months following ACLR. Out of the four parameters identified (eccentric deceleration impulse, concentric impulse, landing impulse and jump height) only the eccentric deceleration impulse significantly decreased between 6 and 9 months post‐ACLR [13].

### Single‐leg DJ

Five cross‐sectional studies compared between individuals following ACLR (*n* = 142 [*f* = 33, *m* = 98]) and healthy controls (*n* = 260 [*f* = 34, *m* = 217]) [32, 39, 51, 55], with one study not reporting participants' sex [37]. Amongst the five studies, eleven unique parameters were identified. Each parameter was reported once, except for peak vGRF, which appeared in two different studies [37, 51]. The parameters that exhibited significant differences between the two groups were jump height [39], vGRF at initial contact [37], reactive strength index (RSI) [39] and reactive strength ratio [39], all favouring the healthy control group. Conversely, the ACLR group demonstrated significantly higher jump height asymmetry [55] and RSI asymmetry [55] values when compared to the control group.

**Figure 5:**
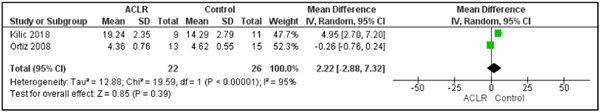
Forest plot comparing peak vertical ground reaction force while performing single‐leg drop‐jump in individuals with and without anterior cruciate ligament reconstruction (ACLR). CI, confidence interval; SD, standard deviation.

However, there were no significant differences observed in peak vGRF symmetry, loading rate symmetry, vGRF at last contact [37] and contact time between the two groups. Notably, peak vGRF showed a significant difference in one study [37] but not in the other [51]. After pooling mean differences, our meta‐analysis revealed no significant difference in peak vGRF between individuals with ACLR and healthy controls during single‐leg DJs (Figure 5). However, it is important to note the varied assessment timings between the two studies (at 6–15 months [37] vs. 86.4 ± 50.4 months [51]) post‐ACLR.

We identified only one longitudinal study assessing single‐leg DJ of 64 athletes at different time points post‐ACLR (8 months and 2 years later). Peak vGRF in involved and uninvolved limbs were not different at 2 years after RTS compared to it at 8 months postsurgery (*p* = 0.08, *p* = 0.18, respectively). No other kinetic parameters were reported [33].

### Double‐leg DJ

Fifteen cross‐sectional studies compared between people following ACLR (*n* = 453 [*f* = 281, *m* = 153]) and healthy controls (*n* = 346 [*f* = 222, *m* = 110]) [8, 9, 17, 19, 22, 31, 32, 41, 42, 46, 48, 53, 61, 63, 64]. Two studies did not report the participants' sex [19, 41]. We identified 21 unique parameters across the 15 studies. Peak vGRF_(normalised)_ was the most frequently used parameter being reported in 11 (73.3%) studies. Peak vGRF_(normalised)_ during the eccentric and concentric phases of jumping were reported in 10 (66.7%) [9, 22, 31, 42, 46, 48, 53, 61, 64, 65] and three (20.0%) [42, 48, 53] studies, respectively. One study reported peak vGRF_(normalised)_ without specifying the phase of the jump [41].

In our meta‐analysis of the 10 studies [9, 22, 31, 42, 46, 48, 53, 61, 64, 65], the involved limb demonstrated significantly lower peak vGRF_(normalised)_ during the eccentric phase of the jump compared to healthy control participants (MD = −0.10 N kg^−1^; *p* = 0.03; 95% CI: [−0.18, −0.01]) with a moderate but insignificant heterogeneity (*I* ^2^ = 35%, *p* = 0.13).

**Figure 6:**
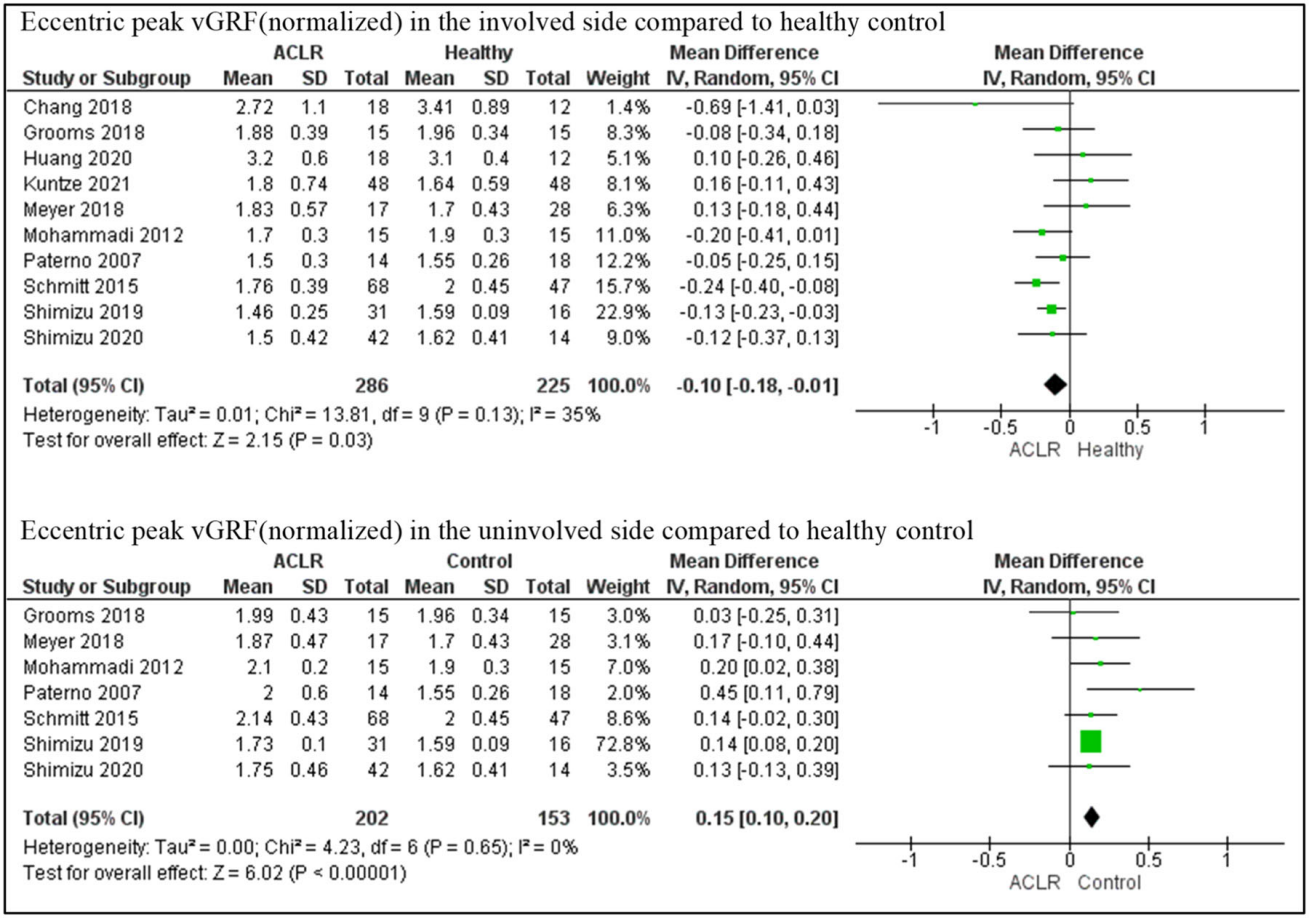
Forest plots comparing eccentric peak vertical ground reaction force (vGRF) in the involved and uninvolved sides while performing double‐leg drop jump in individuals with and without anterior cruciate ligament reconstruction (ACLR). CI, confidence interval; SD, standard deviation.

Furthermore, out of the 10 studies reporting the eccentric peak vGRF_(normalised)_, seven studies provided values both for the involved and non‐involved sides [22, 46, 48, 53, 61, 64, 65]. Interestingly, the uninvolved side of individuals who have undergone ACLR demonstrated greater eccentric peak vGRF_(normalised)_ compared to healthy controls (MD = 0.15 N kg^−1^; *p* < 0.01; 95% CI: [0.10, 0.20]). Importantly, the outcomes of these studies were consistent, with no heterogeneity observed (*I* ^2^ = 0%, *p* = 0.65). (Figure 6).

Concentric peak vGRF_(normalised)_ in the involved and uninvolved sides, eccentric loading rate_(normalised)_ in the involved and uninvolved sides and eccentric loading rate asymmetry, eccentric peak vGRF asymmetry were all not different in the ACLR group compared to the control group (Supporting Information S1: Appendix IV).

Five longitudinal studies assessed DJ in participants with ACLR (*n* = 196 [*f* = 88, *m* = 108]) at different time points [33, 49, 63, 64, 65]. We identified six unique parameters across the studies: (peak vGRF_(normalised)_, peak vGRF_(normalised)_ symmetry index, eccentric peak vGRF_(normalised)_, concentric peak vGRF_(normalised)_, contact time and vGRF impulse). Peak vGRF_(normalised)_ was the most commonly evaluated parameter been reported in all the five studies.

**Figure 7:**
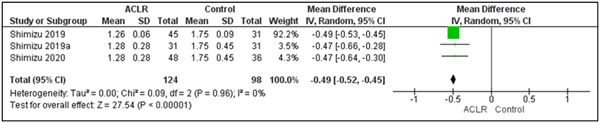
Peak vertical ground reaction force during the stance phase of double‐leg drop jump at 6 months and 3 years postanterior cruciate ligament reconstruction (ACLR). CI, confidence interval; SD, standard deviation.

One study analysed the performance of double‐leg DJ at 8 months post‐ACLR (time to RTS) and 2 years later [33]. For peak vGRF_(normalised)_, involved limb values increased (*p* < 0.01) and uninvolved limb values decreased (*p* < 0.01) from the time of RTS to 2 years later. Accordingly, the peak vGRF_(normalised)_ symmetry index improved at 2 years after RTS (*p* = 0.03) [33]. Another study compared double‐leg DJ performances at 6 and 12 months post‐ACLR and reported extremely high eccentric and concentric peak vGRF_(normalised)_ values [49]. Given these unusually high values, we excluded this study from the meta‐analysis. The final three studies, by the same authors, presented measurements at 6 months and 3 years post‐ACLR in two studies [63, 65] and measurements at 6, 12 months, 2 and 3 years in the third [64]. Interestingly, despite different sample sizes, the mean and SDs at 6 months and 3 years were identical in two out of the three studies. Given the sample size differences between the studies, we could not justify excluding any of the studies and proceeded to cautiously include all three in a meta‐analyses which demonstrated a significantly lower peak vGRF_(normalised)_ during the stance phase of the jump at 6 months compared to 3‐year post‐ACLR (MD = −0.47 N kg^−1^; *p* < 0.01; 95% CI: [−0.52, −0.45]) (Figure 7).

## DISCUSSION

This systematic review provides a rigorous synthesis of evidence and expands on the existing literature, contributing to the on‐going development of this field. The meta‐analyses have yielded valuable insights into the use of force plate parameters to differentiate neuromuscular function between individuals following ACLR and healthy controls during CMJs and DJs. Our findings indicate that specific force plate parameters, such as jump height during single and double‐leg CMJs and peak vGRF normalised during DJs, exhibit significant differences between individuals with and without a history of primary ACLR. Notably, the discriminative ability of these parameters is influenced by factors such as the type of jump (single vs. double‐leg) and the limb side involved. Specifically, jump height was notably lower in individuals post‐ACLR compared to healthy controls. Additionally, eccentric peak vGRF was significantly higher in the uninvolved side and significantly lower in the involved side in individuals post‐ACLR compared to healthy controls. However, our sensitivity analysis revealed heterogeneity in the discriminative ability of other parameters, suggesting the potential need for a combination of parameters to more accurately identify functional deficits post‐ACLR. These findings have important implications for clinical practice and rehabilitation strategies following ACLR.

When interpreting the results of force plate parameters in individuals following ACLR, it is crucial to consider the time of assessment after ACLR. The recovery process following ACLR is dynamic and can vary over time [23]. Therefore, the functional deficits and neuromuscular function observed at 6 months postsurgery may differ from those observed several months later.

While performing single‐leg CMJs, even when assessed at different time points (26.5 ± 6.6 [28] vs. 9.5 ± 2.7 [39] vs. 6.6 ± 1.0 [50] months) post‐ACLR, jump height was the most discriminative parameter. It is a simple and easily interpreted measure that might reflect the capacity to generate power through the kinetic chain [30]. The fact that jump height was consistently different between individuals with and without a history of ACLR highlights the relevance of addressing explosive power deficits in rehabilitation programmes [6]. However, the findings of our meta‐analysis should be taken with caution as only male participants were included in these studies. This may limit the generalisability of findings to female individuals who have a higher risk of ACL injuries [11].

In double‐leg CMJs, multiple parameters, including jump height, concentric and eccentric impulse and several other force measures, demonstrated significant differences. Jump height was notably lower in individuals post‐ACLR compared to healthy controls. Additionally, the concentric impulse was significantly lower in the involved leg, but higher in the uninvolved leg post‐ACLR compared to healthy controls. Both eccentric impulse and concentric impulse demonstrated significantly higher asymmetries in the ACLR group compared to the healthy controls. This provides a more detailed understanding of potential deficits and functional asymmetries in individuals post‐ACLR. These parameters, especially impulse measures, could inform clinicians and researchers about the efficiency of strength generation during jump tasks, which is crucial for safe and effective sports participation and a safe RTS [56, 66].

In the context of DJs, it was found that vGRF during the eccentric phase was discriminative in double‐leg DJs, more so when examining the uninvolved side. Our sensitivity analysis revealed less heterogeneity when studying the uninvolved side compared to the involved side. While it is the same sample of individuals performing exactly the same jump, the increased heterogeneity in the involved side could be related to the type of grafts used and the different rehabilitation protocols that were followed mainly in the involved limb. Vertical GRF is a fundamental parameter in understanding the load absorption capacity of the lower limbs, which is of critical importance in preventing reinjury. However, there was inconsistency regarding the phase of the jump during which the peak vGRF_(normalised)_ values were calculated across studies, and the definitions of those phases, limiting our ability to compare these values accurately. Our longitudinal analysis also revealed significant changes over time post‐ACLR in certain parameters, notably in peak vGRF_(normalised)_ during the stance phase of DJs. This suggests that some aspects of dynamic function improve during the first few years after ACLR, emphasising the potential value of extended rehabilitation and monitoring [21, 34].

The included studies generally had low to moderate methodological quality, as assessed by the Modified DB criteria. Common methodological limitations included partial descriptions of primary confounders, small sample sizes and lack of clarity in the differentiation between participants who chose to participate versus those who did not. These limitations should be addressed in future studies to improve the robustness of findings.

The findings from this systematic review and meta‐analyses bear significant clinical implications for the management of patients following ACLR. The identified force plate parameters, including jump height, concentric and eccentric impulse and vGRF, serve as crucial tools when evaluating neuromuscular function and recovery progress. Their utilisation can guide clinicians in designing more individualised, effective rehabilitation programmes targeting specific functional deficits post‐ACLR. For instance, consistent differences in jump height between ACLR patients and healthy controls underscore the need to incorporate training strategies that enhance explosive power in rehabilitation programmes. Moreover, the fact that some of the identified parameters are responsive to changes over time following ACLR highlights the importance of extended rehabilitation and long‐term monitoring to ensure safe RTSs. Finally, the observed methodological shortcomings in the reviewed studies signal the need for more rigorous research methodologies in the future. We would like to stress the criticality of reporting data sufficiently and precisely to ensure methodological robustness that can be translated into effective practices and policies [18, 44].

This review, however, has some limitations. The lack of standardised methodological protocols across studies while evaluating kinetic parameters during CMJ and DJ may have impacted the results. Additionally, we reported several heterogeneities amongst the studies, particularly, within the individuals following ACLR. While we included studies of participants who are at least 6 months post‐ACLR, we did not account in our analysis for other factors that could have contributed to the heterogeneity such as the graft type, the time since surgery, as well as the rehabilitation protocols followed. However, this systematic review and meta‐analysis has several strengths. The comprehensive search strategy and predefined inclusion and exclusion criteria were implemented to mitigate the risk of overlooking relevant studies ensuring the robustness and thoroughness of the study selection process. The study team consisted of a multidisciplinary group, including individuals with diverse expertise in research methodology, evidence synthesis, orthopaedic surgery, sports and exercise therapy, knee injury rehabilitation, kinesiology and engineering. This diverse range of skills and knowledge minimised ambiguity and uncertainties related to study selection, ensuring a comprehensive approach to the research process.

## CONCLUSION

In conclusion, this review provides a comprehensive overview of the discriminative ability of force plate parameters in individuals post‐ACLR during CMJs and DJs. Future research should strive for improved methodological quality and consider both cross‐sectional and longitudinal designs to monitor changes over time. Moreover, standardising the specific phases of jumps when measurements are taken is necessary to enhance the robustness and the validation of the findings.

## AUTHOR CONTRIBUTIONS

All authors contributed to the study conception and design. Study protocol was prepared by Wasim Labban, revised and approved by all authors. The search keywords were prepared by the study team. An extensive list of search terms was developed, and the literature search was done by Liz Dennett and Wasim Labban. The title and abstract screening, full‐text review, data extraction and quality assessment were performed by Wasim Labban and Thaer Manaseer. The manuscript was prepared by Wasim Labban and was critically revised and edited by all authors. All authors approved the final manuscript.

## CONFLICT OF INTEREST STATEMENT

The authors declare no conflict of interest.

## ETHICS STATEMENT

The authors have nothing to report.

## Supporting information

Supporting information.

## Data Availability

All data generated or analysed during this study are included in this published article (and its Supporting Information files).
