# Supplementary material for: Jumping into recovery: A systematic review and meta‐analysis of discriminatory and responsive force plate parameters in individuals following anterior cruciate ligament reconstruction during countermovement and drop jumps
Source: J Exp Orthop. 2024 Apr 2;11(2):e12018. doi: 10.1002/jeo2.12018 (PMC10986632; doi:10.1002/jeo2.12018)
Supplement: Supplementary file 1 — Supporting information. [file JEO2-11-e12018-s001.docx]

**Appendix I- Search Strategies**

**Ovid MEDLINE(R) ALL**

Date searched: March 13, 2022

Results: 171

1. exp anterior cruciate ligament reconstruction/
2. ((Anterior cruciate ligament or ACL) adj8 (repair or reconstruct* or surgery or post-operativ* or postoperativ*)).mp.
3. 1 or 2
4. (((Bilateral or unilateral or countermovement or counter or squat or drop or vertical or one-leg* or two-leg* or single-leg* or double-leg*) adj4 jump*) or drop land* or jump landing or jump down).mp.
5. (forceplate* or force plate* or force platform* or Kistler or GRF or GRFs or VGRF or VGRFs or pGRF? or ground reaction force* or kinetic* or center of pressure or centre of pressure or centre of mass or center of mass or Reactive strength index or RSImod or impulse or force-development or force-production or force time curve or (jump adj2 (height or duration or phase length)) or flight time or peak force* or limb-impulse* or phase specific or time curve or velocity or between limb difference* or between limb deficit* or ((Leg or legs or limb or limbs or knee or knees or functional or strength or muscle or index or indices or measur*) adj4 (asymmetr* or symmetr*))).mp.
6. 3 and 4 and 5

**Embase 1974 to 2022 March 11  (OVID interface)**

Date searched:March 13, 2022

Results: 183

1. anterior cruciate ligament reconstruction/
2. ((Anterior cruciate ligament or ACL) adj8 (repair or reconstruct* or surgery or post-operativ* or postoperativ*)).mp.
3. (forceplate* or force plate* or force platform* or Kistler or GRF or GRFs or VGRF or VGRFs or pGRF? or ground reaction force* or kinetic* or center of pressure or centre of pressure or centre of mass or center of mass or Reactive strength index or RSImod or impulse or force-development or force-production or force time curve or (jump adj2 (height or duration or phase length)) or flight time or peak force* or limb-impulse* or phase specific or time curve or velocity or between limb difference* or between limb deficit* or ((Leg or legs or limb or limbs or knee or knees or functional or strength or muscle or index or indices or measur*) adj4 (asymmetr* or symmetr*))).mp.
4. (((Bilateral or unilateral or countermovement or counter or squat or drop or vertical or one-leg* or two-leg* or single-leg* or double-leg*) adj4 jump*) or drop land* or jump landing or jump down).mp.
5. (1 or 2) and 3 and 4
6. limit 5 to conference abstracts
7. 5 not 6

**CINAHL Plus with Full Text (EBSCOhose interface)**

Date searched: Mar 13, 2022

Results: 153

Deselect: Apply equivalent subjects

( (MH "Anterior Cruciate Ligament Reconstruction") OR ( (Anterior cruciate ligament or ACL) N8 (repair or reconstruct* or surgery or post-operativ* or postoperativ*)) ) AND ( ((Bilateral or unilateral or countermovement or counter or squat or drop or vertical or one-leg* or two-leg* or single-leg* or double-leg*) N4 jump*) or drop-land* or jump-landing or jump-down ) AND TX ( forceplate* or force-plate* or force-platform* or Kistler or GRF or GRFs or VGRF or VGRFs or pGRF* or ground-reaction-force* or kinetic* or center-of-pressure or centre-of-pressure or centre-of-mass or center-of-mass or Reactive-strength-index or RSImod or impulse or force-development or force-production or force-time-curve or (jump adj2 (height or duration or phase length)) or flight-time or peak-force* or limb-impulse* or phase-specific or time-curve or velocity or between-limb-difference* or between-limb-deficit* or ((Leg or legs or limb or limbs or knee or knees or functional or strength or muscle or index or indices or measur*) N4 (asymmetr* or symmetr*)) )

**SPORTDiscus with Full Text (EBSCOhose interface)**

Date searched: March 13, 2022

Results: 164

( ( (Anterior cruciate ligament or ACL) N8 (repair or reconstruct* or surgery or post-operativ* or postoperativ*)) ) AND ( ((Bilateral or unilateral or countermovement or counter or squat or drop or vertical or one-leg* or two-leg* or single-leg* or double-leg*) N4 jump*) or drop-land* or jump-landing or jump-down ) AND TX ( forceplate* or force-plate* or force-platform* or Kistler or GRF or GRFs or VGRF or VGRFs or pGRF* or ground-reaction-force* or kinetic* or center-of-pressure or centre-of-pressure or centre-of-mass or center-of-mass or Reactive-strength-index or RSImod or impulse or force-development or force-production or force-time-curve or (jump N2 (height or duration or phase length)) or flight-time or peak-force* or limb-impulse* or phase-specific or time-curve or velocity or between-limb-difference* or between-limb-deficit* or ((Leg or legs or limb or limbs or knee or knees or functional or strength or muscle or index or indices or measur*) N4 (asymmetr* or symmetr*)) )

**SCOPUS**

Date searched:March 13, 2022

Results: 198

TITLE-ABS-KEY ( ( anterior-cruciate-ligament OR acl ) W/8 ( repair OR reconstruct* OR surgery OR post-operativ* OR postoperativ* ) ) AND TITLE-ABS-KEY ( ( ( bilateral OR unilateral OR countermovement OR counter OR squat OR drop OR vertical OR one-leg* OR two-leg* OR single-leg* OR double-leg* ) W/4 jump* ) OR drop-land* OR jump-landing OR jump-down ) AND TITLE-ABS-KEY ( forceplate* OR force-plate* OR force-platform* OR kistler OR grf OR grfs OR vgrf OR vgrfs OR pgrf* OR ground-reaction-force* OR kinetic* OR center-of-pressure OR centre-of-pressure OR centre-of-mass OR center-of-mass OR reactive-strength-index OR rsimod OR impulse OR force-development OR force-production OR force-time-curve OR ( jump W/2 ( height OR duration OR phase-length ) ) OR flight-time OR peak-force* OR limb-impulse* OR phase-specific OR time-curve OR velocity OR between-limb-difference* OR between-limb-deficit* OR ( ( leg OR legs OR limb OR limbs OR knee OR knees OR functional OR strength OR muscle OR index OR indices OR measur* ) W/4 ( asymmetr* OR symmetr* ) ) )

**Web of Science Core Collection**

(Indexes=Science Citation Index (CI) Expanded, Social Sciences CI, Arts & Humanities CI, Emerging Sources CI)

Date searched: March13, 2022

Results: 189

TS= ( ( anterior-cruciate-ligament OR acl ) NEAR/8 ( repair OR reconstruct* OR surgery OR post-operativ* OR postoperativ* ) ) AND TS= ( ( ( bilateral OR unilateral OR countermovement OR counter OR squat OR drop OR vertical OR one-leg* OR two-leg* OR single-leg* OR double-leg* ) NEAR/4 jump* ) OR drop-land* OR jump-landing OR jump-down ) AND TS=( forceplate* OR force-plate* OR force-platform* OR kistler OR grf OR grfs OR vgrf OR vgrfs OR pgrf* OR ground-reaction-force* OR kinetic* OR center-of-pressure OR centre-of-pressure OR centre-of-mass OR center-of-mass OR reactive-strength-index OR rsimod OR impulse OR force-development OR force-production OR force-time-curve OR ( jump NEAR/2 ( height OR duration OR phase-length ) ) OR flight-time OR peak-force* OR limb-impulse* OR phase-specific OR time-curve OR velocity OR between-limb-difference* OR between-limb-deficit* OR ( ( leg OR legs OR limb OR limbs OR knee OR knees OR functional OR strength OR muscle OR index OR indices OR measur* ) NEAR/4 ( asymmetr* OR symmetr* ) ) )

**Dissertations and Theses Global (Proquest interface)**

Date searched:March 13, 2022

Results: 28

noft((anterior-cruciate-ligament OR acl) NEAR/8 (repair OR reconstruct* OR surgery OR post-operativ* OR postoperativ*)) AND noft(( ( bilateral OR unilateral OR countermovement OR counter OR squat OR drop OR vertical OR one-leg* OR two-leg* OR single-leg* OR double-leg* ) NEAR/4 jump* ) OR drop-land* OR jump-landing OR jump-down) AND (forceplate* OR force-plate* OR force-platform* OR kistler OR grf OR grfs OR vgrf OR vgrfs OR pgrf* OR ground-reaction-force* OR kinetic* OR center-of-pressure OR centre-of-pressure OR centre-of-mass OR center-of-mass OR reactive-strength-index OR rsimod OR impulse OR force-development OR force-production OR force-time-curve OR ( jump NEAR/2 ( height OR duration OR phase-length ) ) OR flight-time OR peak-force* OR limb-impulse* OR phase-specific OR time-curve OR velocity OR between-limb-difference* OR between-limb-deficit* OR ( ( leg OR legs OR limb OR limbs OR knee OR knees OR functional OR strength OR muscle OR index OR indices OR measur* ) NEAR/4 ( asymmetr* OR symmetr* ) ))

**University of Alberta's Science Direct Journals (Advanced Search)**

Date searched: March 13, 2022

Results: 38

Do not include conference abstracts

*Find articles with these terms*: "ground reaction force" OR kinetic OR "asymmetry index" OR "symmetry index" OR "limb symmetry" OR "limb asymmetry" OR "leg symmetry" OR "Force plate" OR "jump height"

*Title, abstract or author-specified keywords:* "Anterior Cruciate Ligament" AND (repair OR reconstruct OR reconstruction) AND ("drop jump" OR "vertical jump" OR "drop land" OR "landing task" OR "countermovement jump")

**Pubmed Central**

Date searched: March 13, 2022

Results: 74

(("anterior cruciate ligament"[Abstract] or ACL[abstract]) AND (reconstruct[Abstract] OR reconstructed[Abstract] OR reconstruction[Abstract] OR repair[Abstract] OR repaired[Abstract] OR surgery[Abstract] OR post-operative[Abstract] OR postoperative[Abstract]) AND ( vertical-jump[Abstract] OR drop-jump[Abstract] OR drop-land[Abstract] OR land-task[Abstract] OR single-leg-jump[Abstract] OR one-leg-jump[Abstract] OR double-leg-jump[Abstract] OR two-leg-jump[Abstract] OR countermovement-jump[Abstract] OR counter-jump[Abstract] OR vertical-jumps[Abstract] OR drop-jumps[Abstract] OR drop-landing[Abstract] OR landing-task[Abstract] OR landing-tasks[Abstract] OR single-leg-jumps[Abstract] OR one-leg-jumps[Abstract] OR double-leg-jumps[Abstract] OR two-leg-jumps[Abstract] OR countermovement-jumps[Abstract] OR counter-jumps[Abstract])) AND ("ground reaction force" OR "ground reaction forces" OR "force plate" OR "force plates" OR forceplate* OR kinetic OR kinetics OR "asymmetry index" OR "symmetry index" OR "asymmetry indices" OR "symmetry indices" OR "limb symmetr*" OR "limb asymmetr*" OR "leg symmetr*" OR between-limb-difference* OR between-limb-deficit* OR "jump height" OR "Peak force" OR "force development" OR force-production OR force-time-curve OR kistler OR grf OR grfs OR vgrf OR vgrfs OR pgrf* OR reactive-strength-index OR rsimod OR center-of-pressure OR centre-of-pressure OR centre-of-mass OR center-of-mass OR reactive-strength-index OR rsimod OR "concentric impulse" or "eccentric impulse" or "unweighting impulse" or "breaking impulse" or "deceleration impulse" OR limb-impulse* OR "flight time")

**Appendix II – Data Extraction Table**

| Study Characteristics  (author, year, design, country, language) | Sample Characteristics (size, sex, age, graft, time since surgery [in months], physical activity, activity level) | Parameters | Protocol | DB Score |
| --- | --- | --- | --- | --- |
| Single-leg Countermovement Jump | | | |  |
| Giesche 2021 Cross sectional Germany  In English | Control: [n=17, (f=0), (m=17)], age: 28±4 ACLR: [n=10, (f=0), (m=10)], age: 28±4 Graft type: N/R Time since surgery: 63±35 PA (Control): N/R, Level: N/R PA (ACLR): N/R, Level: N/R | -Landing peak vGRF_(normalized)_  -CoP length of path -TTS -Flight time | Sampling frequency: N/R Shoes on/off: N/R Hand position: On hips Warm up: N/R Number of trials: 40 | 11 |
| Holsgaard-Larsen 2014 Cross sectional Denmark  In English | Control: [n=25, (f=0), (m=25)], age: 27.2±5.4 ACLR: [n=23, (f=0), (m=23)], age: 27.2±7.2 Graft type: Hamstring Time since surgery: 26.5±6.6 PA (Control): N/R, Level: N/R PA (ACLR): N/R, Level: N/R | -*Jump Height | Sampling frequency: 1000 Shoes on/off: Off Hand position: On hips Warm up: Reported Number of trials: 3 | 8 |
| Kotsifaki 2022 Cross Sectional Qatar  In English | Control: [n=22, (f=0), (m=22)], age: 28.7±3.8 ACLR: [n=26, (f=0), (m=26)], age: 23.2±3.4 Graft type: HT 10, PT 16 Time since surgery: 9.5±2.7 PA (Control): N/R, Level: Recreational PA (ACLR): N/R, Level: Recreational | -*Jump Height | Sampling frequency: 1000 Shoes on/off: On Hand position: On hips Warm up: Reported  Number of trials: 4 | 9 |
| O'Malley 2018 Cross Sectional Irland  In English | Control: [n=44, (f=0), (m=44)], age: 24.1±3.6 ACLR: [n=118, (f=0), (m=118)], age: 23.6±5.8 Graft type: N/R Time since surgery: 6.6±1.0 PA (Control): Multidirectional sports, Level: N/R PA (ACLR): Multidirectional sports, Level: N/R | -*Jump Height -*Peak power_(normalized)_ | Sampling frequency: 1000 Shoes on/off: N/R Hand position: On hips Warm up: Reported Number of trials: 3 | 10 |
| Double-leg Countermovement Jump | | | |  |
| Castanharo 2011 Cross Sectional Brazil  In English | Control: [n=17, (f=0), (m=17)], age: 26±4 ACLR: [n=12, (f=0), (m=12)], age: 28±8 Graft type: unilateral semitendinous-gracilis tendon autograft, with no more than 25% of the meniscus removed Time since surgery: 37±9 PA (Control): N/R, Level: Recreational sports activity PA (ACLR): N/R, Level: Recreational sports activity | -Concentric peak vGRF_(normalized)_ -Landing Peak vGRF_(normalized)_ | Sampling frequency: 1080 Hz Shoes on/off: On Hand position: On chest Warm up: N/R Number of trials: 6 | 8 |
| Collings 2021 Cross Sectional Australia  In English | Control: [n=198, (f=198), (m=0)], age: 20.3±8.6 ACLR: [n=24, (f=198), (m=0)], age: 23.1±5.1 Graft type: 21 hamstring, 1 patellar, and 2 quadriceps Time since surgery: N/R  PA (Control): Football, Soccer and Rugby, Level: elite PA (ACLR): Football, Soccer and Rugby, Level: elite | -Peak GRF_(normalized)_ (Take-off) -Peak GRF_(normalized)_ (Landing) -Take-off Impulse_(normalized)_ | Sampling frequency: 1000 Hz Shoes on/off: N/R Hand position: On hips Warm up: Reported Number of trials: 3 | 11 |
| Jordan 2018 Cross Sectional Canada  In English | Control: [n=24, (f=12), (m=12)], age: 20.9±3.7 ACLR: [n=12, (f=12), (m=6)], age: 26.7±3.8 Graft type: N/R Time since surgery: 48, range (2y-8y) PA (Control): Skiing, Level: Mixed PA (ACLR): Skiing, Level: Mixed | -Contraction time -*Jump height -*Relative peak power_(normalized)_ -Relative mean power -*Velocity max -*Relative force at max velocity -Relative force at peak power_(normalized)_ -Velocity at peak power_(normalized)_  -Impulse eccentric asymmetry  -*Impulse concentric asymmetry | Sampling frequency: 1500 Shoes on/off: N/R Hand position: On hips Warm up: Reported Number of trials: 5 | 8 |
| Jordan 2015 Cross Sectional Canada  In English | Control: [n=9, (f=4), (m=5)], age: 22.3±2.3 ACLR: [n=9, (f=4), (m=4)], age: 26.8±4.4 Graft type: N/R Time since surgery: 26.2±11.8 PA (Control): Skiing, Level: Elite PA (ACLR): Skiing, Level: Elite | -Impulse eccentric -Impulse concentric  -Impulse eccentric asymmetry  -*Impulse concentric asymmetry | Sampling frequency: 500 Shoes on/off: N/R Hand position: On hips Warm up: Reported Number of trials: 10 | 12 |
| Krafft 2017 Cross sectional data of a longitudinal study Germany  In English | Control: [n=20, (f=N/R), (m=N/R)], age: 33.3±13.4 ACLR: [n=20, (f=N/R), (m=N/R)], age: 32±13.3 Graft type: combined semitendinosus and gracilis autograft, via the single-bundle technique Time since surgery: 6.31±0.64 PA (Control): N/R, Level: Recreational PA (ACLR): N/R, Level: Recreational | -*Jump height -*Impulse eccentric LSI -*Impulse concentric LSI | Sampling frequency: 1000 Shoes on/off: N/R Hand position: On hips Warm up: N/R Number of trials: 3 | 8 |
| Miles 2019 Cross Sectional Ireland  In English | Control: [n=22, (f=0), (m=22)], age: 23.1±3.4 ACLR: [n=44, (f=0), (m=44)], age: 24.8±4.6 Graft type: HT 22, PT 22 Time since surgery: N/R±N/R PA (Control): (Gaelic football and hurling; 66%), soccer (24%), and rugby, Level: N/R PA (ACLR): (Gaelic football and hurling; 66%), soccer (24%), and 10rugby, Level: N/R | -*Impulse_(normalized)_ eccentric -*Impulse_(normalized)_ concentric -*Impulse_(normalized)_ landing -*Jump Height | Sampling frequency: 1000 Shoes on/off: N/R Hand position: On hips  Warm up: Reported  Number of trials: 3 | 11 |
| Read 2020 Cross Sectional Qatar  In English | Co11ntrol: [n=204, (f=0), (m=204)], age: 24.4±4.7 ACLR: [n=124, (f=0), (m=124)], age: 23.83±6.13 Graft type: N/R Time since surgery: <6 (6-9) >9± PA (Control): Soccer, Level: Elite PA (ACLR): Soccer, Level: Elite | -*Jump height -*Peak power -*Impulse concentric -*Impulse concentric asymmetry -Impulse eccentric deceleration -*Impulse eccentric deceleration asymmetry -*Concentric peak vGRF -*Concentric peak vGRF asymmetry -*Eccentric deceleration RFD -*Eccentric deceleration RFD asymmetry -*Eccentric mean GRF -*Eccentric mean GRF asymmetry -*Peak vGRF (Landing) -*Peak vGRF (Landing) asymmetry | Sampling frequency: 1000 Shoes on/off: N/R Hand position: On hips Warm up: Reported Number of trials: 3 | 10 |
| Costley 2021 Longitudinal Ireland  In English | Control: [n=44, (f=44), (m=0)], age: 4.6±N/A ACLR: [n=N/A, (f=44), (m=0)], age: N/A±24.8 Graft type: N/A Time since surgery: N/R±5-7 months PA (Control): multidirectional field sports, Level: Recreational PA (ACLR): multidirectional field sports, Level: Recreational | -*Eccentric deceleration impulse_(normalized)_  -Concentric impulse_(normalized)_  -Landing impulse_(normalized)_ -Jump height | Sampling frequency: 1000 hz Shoes on/off: N/R Hand position: On hips Warm up: Reported Number of trials: 3 | 11 |
| Single-leg Drop Jump | | | |  |
| Huang 2021 Cross Sectional USA  In English | Control: [n=19, (f=19), (m=0)], age: 21.1±3.3 ACLR: [n=19, (f=19), (m=0)], age: 19.9±1.2 Graft type: N/R Time since surgery: 35.1±13.7 PA (Control): soccer, basketball, and handball, Level: Recreational PA (ACLR): soccer, basketball, and handball, Level: Recreational | -Eccentric Peak vGRF_(normalized)_ symmetry -Eccentric loading rate_(normalized)_ symmetry | Sampling frequency: 1500 Shoes on/off: On Hand position: Free Warm up: N/R Number of trials: 3 | 10 |
| Kilic 2018 Cross Sectional Turkey  In English | Control: [n=9, (f=N/R), (m=N/R)], age: 22.2±2.5 ACLR: [n=11, (f=N/R), (m=N/R)], age: 23.1±3.6 Graft type: patellar tendon Time since surgery: ±range (6-15) PA (Control): Soccer, Level: Recreational PA (ACLR): Soccer, Level: Recreational | -*vGRF at initial contact -*Peak vGRF -vGRF at last contact | Sampling frequency: 1000 Shoes on/off: On Hand position: On hips Warm up: Reported Number of trials: 3 | 10 |
| Kotsifaki 2022 Cross Sectional Qatar  In English | Control: [n=22, (f=0), (m=22)], age: 28.7±3.8 ACLR: [n=26, (f=0), (m=26)], age: 23.2±3.4 Graft type: HT 10, PT 16 Time since surgery: 9.5±2.7 PA (Control): N/R, Level: Recreational PA (ACLR): N/R, Level: Recreational | -*Jump height -*RSI -*RSR -Contact time | Sampling frequency: 1000 Shoes on/off: On Hand position: On hips Warm up: Reported Number of trials: 4 | 9 |
| Ortiz 2008 Cross Sectional Puerto Rico  In English | Control: [n=15, (f=15), (m=0)], age: 24.6±2.6 ACLR: [n=14, (f=15), (m=0)], age: 25.4±3.1 Graft type: N/R Time since surgery: 86.4±50.4 PA (Control): Jogging, running, and weight lifting, Level: Recreational PA (ACLR): Jogging, running, and weight lifting, Level: Recreational | -Peak vGRF | Sampling frequency: 1000 Shoes on/off: On Hand position: Free Warm up: Reported Number of trials: 6 | 9 |
| Read 2020 Cross Sectional Qatar  In English | Control: [n=195, (f=0), (m=195)], age: 24.7±4.3 ACLR: [n=72, (f=0), (m=72)], age: 24.2±5.1 Graft type: N/R Time since surgery: 9±N/R PA (Control): Soccer, Level: N/R PA (ACLR): Soccer, Level: N/R | -*Jump height asymmetry -*RSI asymmetry | Sampling frequency: 1000 Shoes on/off: N/R Hand position: On hips Warm up: Reported Number of trials: 3 | 11 |
| Ithurburn 2019 Longitudinal USA  In English | Control: [n=64, (f=20), (m=N/A)], age: 3.3±N/A ACLR: [n=N/A, (f=20), (m=44)], age: N/A±17 Graft type: N/A Time since surgery: N/R±7.8 PA (Control): N/R, Level: Recreational PA (ACLR): N/R, Level: N/R | -*Peak vGRF_(normalized)_ | Sampling frequency: 1200 Shoes on/off: On Hand position: Free  Warm up: Reported Number of trials: 3 | 8 |
| Double-leg Drop Jump | | | |  |
| Chang 2018 Cross Sectional South Korea  In English | Control: [n=12, (f=12), (m=0)], age: 21±2.6 ACLR: [n=18, (f=12), (m=0)], age: 19.9±1.2 Graft type: N/R Time since surgery: 35.2±13.2 PA (Control): N/R, Level: N/R PA (ACLR): N/R, Level: N/R | -Eccentric peak vGRF_(normalized)_ | Sampling frequency: 1560 Shoes on/off: On Hand position: N/R Warm up: Reported Number of trials: 3 | 10 |
| Chang 2020 Cross Sectional South Korea  In English | Control: [n=12, (f=12), (m=0)], age: 21±2.6 ACLR: [n=18, (f=12), (m=0)], age: 19.9±1.2 Graft type: N/R Time since surgery: 35.2±13.2 PA (Control): N/R, Level: N/R PA (ACLR): N/R, Level: N/R | -Eccentric peak vGRF_(normalized)_ symmetry  -Eccentric loading rate_(normalized)_ symmetry | Sampling frequency: 1560 Shoes on/off: On Hand position: N/R Warm up: Reported Number of trials: 3 | 10 |
| Ford 2016 Cross Sectional USA  In English | Control: [n=57, (f=42), (m=15)], age: 17.2±2.5 ACLR: [n=101, (f=42), (m=37)], age: 16.7±3 Graft type: N/R Time since surgery: 8.3±2.5 PA (Control): N/R, Level: N/R PA (ACLR): N/R, Level: N/R | -*Absolute initial contact timing differences between landing sides in (ms) | Sampling frequency: 1200 Shoes on/off: N/R Hand position: Free Warm up: N/R Number of trials: 2 | 9 |
| Funk 2016 Cross Sectional USA  In English | Control: [n=11, (f=N/R), (m=N/R)], age: 21.1±2 ACLR: [n=12, (f=N/R), (m=N/R)], age: 20.7±1.3 Graft type: N/R Time since surgery: Less than 5 years±N/R PA (Control): N/R, Level: N/R PA (ACLR): N/R, Level: N/R | -Eccentric loading rate_(normalized)_  -Eccentric vGRF_(normalized)_  -Eccentric hGRF_(normalized)_ | Sampling frequency: 1400 Shoes on/off: N/R Hand position: N/R Warm up: Reported Number of trials: 3 | 8 |
| Grooms 2018 Cross Secational USA  In English | Control: [n=15, (f=8), (m=7)], age: 23.2±3.5 ACLR: [n=15, (f=8), (m=7)], age: 21.4±2.6 Graft type: 13 Hamstring, 2 patellar Time since surgery: 36.2±26.5 PA (Control): N/R, Level: Recreational PA (ACLR): N/R, Level: Recreational | -*Eccentric peak vGRF_(normalized)_ | Sampling frequency: N/R Shoes on/off: N/R Hand position: Free Warm up: N/R Number of trials: 3 | 11 |
| Huang 2020 Cross Sectional USA  In English | Control: [n=16, (f=16), (m=0)], age: 21±2.6 ACLR: [n=19, (f=16), (m=0)], age: 19.9±1.2 Graft type: N/R Time since surgery: 35.1±13.7 PA (Control): soccer, basketball, and handball, Level: Recreational PA (ACLR): soccer, basketball, and handball, Level: Recreational | -Eccentric peak vGRF_(normalized)_  -Eccentric peak pGRF_(normalized)_  -Time to peak vGRF  -Time to peak pGRF | Sampling frequency: 1560 Shoes on/off: On Hand position: Free Warm up: N/R Number of trials: 3 | 10 |
| Huang 2021 Cross Sectional USA  In English | Control: [n=19, (f=19), (m=0)], age: 21.1±3.3 ACLR: [n=19, (f=19), (m=0)], age: 19.9±1.2 Graft type: N/R Time since surgery: 35.1±13.7 PA (Control): soccer, basketball, and handball, Level: Recreational PA (ACLR): soccer, basketball, and handball, Level: Recreational | -Eccentric peak vGRF_(normalized)_ asymmetry  - Eccentric loading rate_(normalized)_ asymmetry | Sampling frequency: 1560 Shoes on/off: On Hand position: Free Warm up: N/R Number of trials: 3 | 10 |
| Krysak 2019 Cross Sectional  USA  In English | Control: [n=3, (f=N/R), (m=N/R)], age: 20±2 ACLR: [n=7, (f=N/R), (m=N/R)], age: 20.86±1.86 Graft type: N/R Time since surgery: N/R±N/R PA (Control): N/R, Level: N/R PA (ACLR): N/R, Level: N/R | -Peak vGRF_(normalized)_ | Sampling frequency: 1000 Shoes on/off: On Hand position: Frees Warm up: N/R Number of trials: 5 | 9 |
| Kuntze 2021 Cross Sectional  USA  In English | Control: [n=48, (f=32), (m=16)], age: Median 22±Range (18-26) ACLR: [n=48, (f=32), (m=16)], age: Median 23±Range (18-26) Graft type: N/R Time since surgery: N/R±N/R PA (Control): Several, Level: N/R PA (ACLR): Several, Level: N/R | -Eccentric peak vGRF_(normalized)_  -*Eccentric peak mlGRF_(normalized)_ -Concentric peak vGRF_(normalized)_ -Concentric peak mlGRF_(normalized)_ | Sampling frequency: 2400 Shoes on/off: Off Hand position: N/R Warm up: N/R Number of trials: 10 | 12 |
| Meyer 2018 Cross Sectional  Luxembourg  In English | Control: [n=28, (f=14), (m=14)], age: 24.5±6.8 ACLR: [n=17, (f=14), (m=12)], age: 25.4±4.1 Graft type: HT 11, PT 6 Time since surgery: 8.9±1.3 PA (Control): N/R, Level: N/R PA (ACLR): N/R, Level: N/R | -*Eccentric peak vGRF_(normalized)_ | Sampling frequency: 1000 Shoes on/off: On Hand position: On hips Warm up: Reported Number of trials: 3 | 9 |
| Mohammadi 2012 Cross Sectional  Iran  In English | Control: [n=30, (f=6), (m=24)], age: 24.8±2.4 ACLR: [n=30, (f=6), (m=22)], age: 25±2.7 Graft type: HT 17, PT 13 Time since surgery: 8.4±1.8 PA (Control): soccer/basketball, Level: Recreational PA (ACLR): soccer/basketball, Level: Recreational | -*CoP AP displacement -*CoP AP velocity -*CoP ML displacement -*CoP ML velocity -*CoP mean velocity -Eccentric peak vGRF_(normalized)_ -*Concentric peak vGRF_(normalized)_ -Eccentric loading rate_(normalized)_ | Sampling frequency: 1200 Shoes on/off: N/R Hand position: Free Warm up: N/R Number of trials: N/R | 8 |
| Paterno 2007 Cross Sectional USA  In English | Control: [n=18, (f=18), (m=0)], age: 20±1.2 ACLR: [n=14, (f=18), (m=0)], age: 20.7±2.5 Graft type: PT Time since surgery: 27.4±13.8 PA (Control): N/R, Level: Recreational PA (ACLR): N/R, Level: Recreational | -Eccentric Peak vGRF_(normalized)_ -*Concentric peak vGRF_(normalized)_ -Eccentric loading rate_(normalized)_ | Sampling frequency: N/R Shoes on/off: N/R Hand position: Free Warm up: N/R Number of trials: 3 | 8 |
| Schmitt 2015 Cross Sectional USA  In English | Control: [n=47, (f=32), (m=15)], age: 17±2.3 ACLR: [n=68, (f=32), (m=22)], age: 17.5±2.8 Graft type: PT 31, HS 31, Allo 6 Time since surgery: 8.2±2.1 PA (Control): Several sports, Level: Mix PA (ACLR): Several sports, Level: Mix | -*Eccentric peak vGRF_(normalized)_  -Eccentric loading rate_(normalized)_ | Sampling frequency: 1200 Shoes on/off: N/R Hand position: Free Warm up: N/R Number of trials: 3 | 10 |
| Shimizu 2020 Cross Sectional data of longtudinal study USA  In English | Control: [n=14, (f=5), (m=9)], age: 31.4±4.9 ACLR: [n=36, (f=5), (m=20)], age: 31.5±7.6 Graft type: HS 24, allograft 12 Time since surgery: 6, 12, 24, 36±N/A PA (Control): N/R, Level: N/R PA (ACLR): N/R, Level: N/R | -Eccentric peak vGRF_(normalized)_ | Sampling frequency: 1000 Shoes on/off: N/R Hand position: Free Warm up: N/R Number of trials: 3 | 10 |
| Shimizu 2019 Cross Sectional data of longtudinal study USA  In English | Control: [n=16, (f=6), (m=10)], age: 31.7±1.3 ACLR: [n=31, (f=6), (m=17)], age: 31.3±1.4 Graft type: HS 22, allograft 9 Time since surgery: 6, 36±N/R PA (Control): N/R, Level: N/R PA (ACLR): N/R, Level: N/R | -*Eccentric peak vGRF_(normalized)_  -vGRF Impulse | Sampling frequency: 1000 Shoes on/off: N/R Hand position: Free Warm up: N/R Number of trials: 3 | 10 |
| Ithurburn 2019 Longitudinal USA  In English | Control: [n=N/A, (f=N/A), (m=N/A)], age: N/A±N/A ACLR: [n=64, (f=N/A), (m=20)], age: 17±3.3 Graft type: HS 39, PT 20, Allo 5 Time since surgery: 7.8±2.1 PA (Control): N/R, Level: N/R PA (ACLR): N/R, Level: Recreational | -*Peak vGRF_(normalized)_ asymmetry -*Peak vGRF_(normalized)_ | Sampling frequency: 1200 Shoes on/off: On Hand position: Free Warm up: N/R Number of trials: 3 | 8 |
| Moya-Angeler 2017 Longitudinal USA  In English | Control: [n=N/A, (f=N/A), (m=N/A)], age: N/A±N/A ACLR: [n=74, (f=N/A), (m=34)], age: 34±9 Graft type: N/R Time since surgery: 6±0 PA (Control): N/R, Level: N/R PA (ACLR): N/R, Level: N/R | -*Eccentric peak vGRF_(normalized)_ -*Concentric peak vGRF_(normalized)_ -*Contact time | Sampling frequency: N/R Shoes on/off: N/R Hand position: N/R Warm up: N/R Number of trials: N/R | 7 |
| Shimizu 2019 Longitudinal USA  In English | Control: [n=N/A, (f=N/A), (m=N/A)], age: N/A±N/A ACLR: [n=31, (f=N/A), (m=17)], age: 31.3±7.8 Graft type: HS 23, allograft 8 Time since surgery: 6, 36±N/R PA (Control): N/R, Level: N/R PA (ACLR): N/R, Level: N/R | -*Peak vGRF_(normalized)_ | Sampling frequency: 1000 Shoes on/off: N/R Hand position: Free Warm up: N/R Number of trials: 3 | 10 |
| Shimizu 2020 Longitudinal USA  In English | Control: [n=14, (f=5), (m=9)], age: 31.4±4.9 ACLR: [n=36, (f=5), (m=20)], age: 31.5±7.6 Graft type: HS 24, allograft 12 Time since surgery: 6, 12, 24, 36 PA (Control): N/R, Level: N/R PA (ACLR): N/R, Level: N/R | -*Peak vGRF_(normalized)_ | Sampling frequency: 1000 Shoes on/off: N/R Hand position: Free Warm up: N/R Number of trials: 3 | 10 |
| Shimizu 2019 Longitudinal USA  In English | Control: [n=16, (f=6), (m=10)], age: 31.7±1.3 ACLR: [n=31, (f=6), (m=17)], age: 31.3±1.4 Graft type: HS 22, allograft 9 Time since surgery: 6, 36±N/R PA (Control): N/R, Level: N/R PA (ACLR): N/R, Level: N/R | -*Peak vGRF_(normalized)_ - vGRF Impulse | Sampling frequency: 1000 Shoes on/off: N/R Hand position: Free Warm up: N/R Number of trials: 3 | 10 |
| * significant at p<0.05; N/R: Not Reported  ACLR: Anterior Cruciate Ligament Reconstruction; vGRF: vertical ground reaction force; CoP: center of pressure; TTS: time to stabilization; LSI: limb symmetry index; RSI: reactive strength index, RSR: reactive strength ratio. | | | | |

**Appendix III – Parameters operationalization across the studies**

| Author | Parameters | Operationalization |
| --- | --- | --- |
| Castanharo 2011 | Concentric peak vGRF_(normalized)_ | Specified in the paper as the peak vGRF during the impulsion phase of the jump. Ground reaction forces were normalized to body weight. |
|  | Landing Peak vGRF_(normalized)_ | Ground reaction forces were normalized to body weight. |
| Chang 2018 | Eccentric peak vGRF_(normalized)_ | vGRF at either IC (vGRF >10 N) or the peak value between IC and the time of peak knee flexion. Ground reaction forces were normalized to body weight. |
| Chang 2020 | Eccentric peak vGRF_(normalized)_ symmetry | All dependent variables were identified at initial contact (vGRF>10 N) and/or the peak value during the landing phase (time from initial ground contact to peak knee flexion). Ground reaction forces were normalized to body weight. |
|  | Eccentric loading rate_(normalized)_ symmetry | Loading rate calculation not operationalized. Symmetry: (ACLR: reconstructed limb — non-reconstructed limb, healthy: non-dominant limb — dominant limb). |
| Collings 2021 | Peak GRF_(normalized)_ (Take-off) | Take-off included the eccentric countermovement and concentric propulsion phase. GRFs were normalized to body weight. |
|  | Peak GRF_(normalized)_ (Landing) | Landing was defined from initial ground contact until returning to a standing position. GRFs were normalized to body weight. |
|  | Take-off Impulse_(normalized)_ | Calculated as the area under the force-time curve above standing bodyweight force. GRFs were normalized to body weight. |
| Costley 2021 | Eccentric deceleration impulse_(normalized)_ | Eccentric deceleration phase: from maximal downwards velocity to zero velocity. Impulses were derived by integration of force–time curves. GRFs were normalized to body mass. |
|  | Concentric impulse_(normalized)_ | Concentric phase: from zero velocity to take-off. Impulses were derived by integration of force–time curves. GRFs were normalized to body mass. |
|  | Landing impulse_(normalized)_ | Landing phase: from landing to zero velocity. Impulses were derived by integration of force–time curves. GRFs were normalized to body mass. |
|  | Jump height | The impulse–momentum relationship was used to calculate vertical velocity of the centre of mass at the instant of CMJ take-off (Linthorne, 2001), enabling the determination of peak CMJ height |
| Ford 2016 | Absolute initial contact timing differences between landing sides | Initial contact was as the time that the unfiltered vertical ground reaction force first exceeded 10 N. The absolute time difference between the initial contacts for each leg was calculated and compared between ACLR and control groups. |
| Funk 2016 | Eccentric loading rate_(normalized)_ | Rate of loading was calculated from the time of initial vertical ground reaction force to the time to peak force. Ground reaction forces were normalized to body weight. |
|  | Eccentric vGRF_(normalized)_ | GRFs were normalized to body weight. |
|  | Eccentric hGRF_(normalized)_ | GRFs were normalized to body weight. |
| Giesche 2021 | Landing peak vGRF_(normalized)_ | GRFs were normalized to body weight. |
|  | CoP length of path | During the first 2.5 sec upon landing |
|  | TTS | Estimated relative to the whole standing period of 10 sec after landing. A stable stance is assumed as soon as the sequential average no longer exceeds the threshold of 0.25 SD of the overall mean ground vertical force. |
|  | Flight time | Not operationalized |
| Grooms 2018 | Eccentric peak vGRF_(normalized)_ | Initial contact of each limb was defined as the point when the vertical ground reaction force first exceeded 20 N. The landing phase was defined as the period from initial contact to peak knee flexion. Ground reaction forces were normalized to body weight. |
| Holsgaard-Larsen 2014 | Jump Height | Calculated from the vertical velocity of the body center of mass at take-off, the latter derived by calculation of the kinetic impulse during the entire take-off phase. Specifically, vertical BCM velocity was obtained by time integration of the instantaneous acceleration signal ([Fz / m] − g, where m = body mass and g = 9.81 m/s. |
| Huang 2020 | Eccentric peak vGRF_(normalized)_ | The time when the vGRF >10 Newton was identified as initial contact. Peak vGRF were calculated during the initial 100 ms after inintial contact |
|  | Eccentric peak pGRF_(normalized)_ | Peak posterior ground reaction forces were calculated during the initial 100 ms after inintial contact |
|  | Time to peak vGRF | Time from initial contact to peak vGRF |
|  | Time to peak pGRF | Time from initial contact to peak posterior ground reaction forces |
| Huang 2021 | Eccentric peak vGRF_(normalized)_ asymmetry | During the initial 100 milliseconds after IC, which was defined as the time when the vGRF exceeded 10 N. Asymmetry was calculated as the ACLR limb minus the uninvolved limb for the ACLR group and the nondominant limb minus the dominant limb for the control group. GRFs were normalized to body mass |
|  | Eccentric loading rate_(normalized)_ asymmetry | Loading rate was calculated as the peak vGRF divided by the time from IC to peak vGRF. Asymmetry was calculated as the ACLR limb minus the uninvolved limb for the ACLR group and the nondominant limb minus the dominant limb for the control group. |
| Ithurburn 2019 | Peak vGRF_(normalized)_ asymmetry | Forces were normalized to body weight.  (Involved limb value /uninvolved limb value) x 100%. GRFs were normalized to body weight |
|  | Peak vGRF_(normalized)_ | Forces were normalized to body weight. |
| Jordan 2018 | Contraction time | Determined from the onset of the vertical takeoff (start of the jump) to the instant of ground toe-off |
|  | Jump height | Jump Height = Take-off Velocity^2^/(2g) |
|  | Relative peak power_(normalized)_ | Not operationalized |
|  | Relative mean power_(normalized)_ | Not operationalized |
|  | Velocity max | Not operationalized |
|  | Relative force_(normalized)_ at max velocity | Normalized to body mass |
|  | Relative force_(normalized)_ at peak power | Normalized to body mass |
|  | Velocity at peak power_(normalized)_ | Not operationalized |
|  | Impulse eccentric asymmetry | Integration of the force–time curve over the eccentric deceleration phase duration. Asymmetry was calculated as (Uninjured limb eccentric impulse - ACLR limb eccentric impulse)/(Maximum of left and right eccentric impulse) x 100 |
|  | Impulse concentric asymmetry | Integration of the force–time curve over the concentric phase duration. Asymmetry was calculated as (Uninjured limb eccentric impulse - ACLR limb eccentric impulse)/(Maximum of left and right eccentric impulse) x 100 |
| Jordan 2015 | Impulse eccentric | Integration of the force–time curve over the eccentric deceleration phase duration. |
|  | Impulse concentric | Integration of the force–time curve over the concentric phase duration. |
|  | Impulse eccentric asymmetry | (Uninjured limb eccentric impulse - ACLR limb eccentric impulse)/(Maximum of left and right eccentric impulse) x 100 |
|  | Impulse concentric asymmetry | (Uninjured limb concentric impulse - ACLR limb concentric impulse)/(Maximum of left and right concentric impulse) x 100 |
| Kilic 2018 | vGRF at initial contact | Initial contact phase was defined as the instant where the force plate reported values greater than 20 N. Forces were normalized to body weight. |
|  | Peak vGRF | Forces were normalized to body weight. |
|  | vGRF at last contact | the greatest force value after moment of jump. Forces were normalized to body weight. |
| Kotsifaki 2022 | Jump Height | Measured as the vertical displacement of the centre of mass from toe off to the maximum height of the centre of mass |
|  | RSI | The jump height in a drop jump, divided by the contact time |
|  | RSR | The flight time of the jump divided by the contact time |
|  | Contact time (s) | Not operationalized |
| Krafft 2017 | Jump height | Not operationalized |
|  | Impulse eccentric LSI | The deceleration impulses were measured during landing. LSIs were calculated for all parameters by the related discrete values of the injured leg divided by the uninjured leg in the ACL subjects and by the non-dominant leg divided by the dominant leg in the control subjects, respectively |
|  | Impulse concentric LSI | The acceleration impulses were measured during take-off. LSIs were calculated for all parameters by the related discrete values of the injured leg divided by the uninjured leg in the ACL subjects and by the non-dominant leg divided by the dominant leg in the control subjects, respectively |
| Krysak 2019 | Peak vGRF_(normalized)_ | Forces were normalized to body weight. The stance phase (initial contact to toe-off), with initial contact and toe-off representing the instants when the vertical ground reaction force (vGRF) first exceeded or fell below 10N |
| Kuntze 2021 | Eccentric peak vGRF_(normalized)_ | Initial contact was identified using vGRF data for each force plate with a cutoff of 5% of the peak vGRF. The duration of the support phase of the DJ was determined from the time either leg first contacted the ground (ie, initial contact) until the first time either leg first left the ground (ie, toe-off). Forces were normalized to body weight. |
|  | Eccentric peak mlGRF_(normalized)_ | Forces were normalized to body weight. |
|  | Concentric peak vGRF_(normalized)_ | Forces were normalized to body weight. |
|  | Concentric peak mlGRF_(normalized)_ | Forces were normalized to body weight. |
| Meyer 2018 | Eccentric peak vGRF_(normalized)_ | Initial contact and take-off events were determined based on a 10 N threshold from the vertical ground reaction force vector. The eccentric landing phase defined as initial contact to maximal knee flexion angle. Forces were normalized to body weight. |
| Miles 2019 | Impulse_(normalized)_ eccentric | Take‐off was defined as the first instant the sum of GRFv on both force platforms was <10 N and landing was defined as the first instant the sum of GRFv on both force platforms was >10 N after take‐off. CoM vertical velocity was used to define phases of interest: The eccentric deceleration phase was defined as the time interval from maximum negative velocity to zero velocity (lowest CoM position). Impulse was calculated as integral of the force‐time curve and divided by body mass. |
|  | Impulse_(normalized)_ concentric | the concentric phase was defined from zero velocity to the instant of take‐off. Impulse was calculated as integral of the force‐time curve and divided by body mass. |
|  | Impulse_(normalized)_ landing | the landing phase was defined as the time interval from landing to zero velocity (lowest CoM position). Impulse was calculated as integral of the force‐time curve and divided by body mass |
|  | Jump Height | Jump height was calculated from the vertical velocity of the center of body mass (CoM) at take‐off, as derived from the impulse‐momentum relationship |
| Mohammadi 2012 | CoP AP displacement | Not operationalized |
|  | CoP AP velocity | Not operationalized |
|  | CoP ML displacement | Not operationalized |
|  | CoP ML velocity | Not operationalized |
|  | CoP mean velocity | Not operationalized |
|  | Eccentric peak vGRF_(normalized)_ | participants were asked to drop off a 40-cm platform and immediately jump as high as they can (takeoff phase) and then contact the center of the force plate again (landing phase) |
|  | Concentric peak vGRF_(normalized)_ | participants were asked to drop off a 40-cm platform and immediately jump as high as they can (takeoff phase) and then contact the center of the force plate again (landing phase) |
|  | Eccentric loading rate_(normalized)_ | Peak vGRF normalized to body weight divided by time to reach peak vGRF |
| Moya-Angeler 2017 | Eccentric peak vGRF_(normalized)_ | Mentioned in the paper as fallen maximum vertical force. According the graph, it means eccentric peak vGRF. Forces were normalized to body weight. |
|  | Concentric peak vGRF_(normalized)_ | Mentioned in the paper as impulse maximum vertical force. Operated on the graph as concentric peak vGRF. Forces were normalized to body weight. |
|  | Contact time | Not operated |
| O'Malley 2018 | Jump Height | Jump height was determined using the impulse-momentum relationship |
|  | Peak power_(normalized)_ | Peak power was measured and normalized to body weight during the propulsion phase of the single leg CMJ. Peak power was selected due to its reported relationship to jump height. |
| Ortiz 2008 | Peak vGRF_(normalized)_ | The ground-contact phase: from initial contact as identified by the force plate to push off from the force plate into the vertical jump. Forces were normalized to body weight. |
| Paterno 2007 | Eccentric Peak vGRF_(normalized)_ | Eccentric: Take off phase. Forces were normalized to body weight. |
|  | Concentric peak vGRF_(normalized)_ | Concentric: Landing phase. Forces were normalized to body weight. |
|  | Eccentric loading rate_(normalized)_ | loading rate during landing was calculated as the peak vGRF normalized to body weight divided by time to reach peak vGRF |
| Read 2020 | Jump height asymmetry | Asymmetry calculation was not operationalized. Jump height was calculated using the athletes centre of mass velocity via the following equation: (COM velocity)^2^ / (9.81 x 2). |
|  | RSI asymmetry | Asymmetry calculation was not operationalized. Reactive strength index (RSI) was quantified using the equation jump height/ground contact time. contact time was defined as the time from which the vGRF exceeded 20 N to the instant of take-off |
| Read 2020a | Jump height | The initiation of the jump was defined by a 20-N change from body weight calculated during the quiet standing period. Jump height was calculated from the impulse-momentum relationship–derived take-off velocity and equation of constant acceleration |
|  | Peak power | Not operationalized |
|  | Impulse concentric | Concentric phase, from zero velocity to the instant of take-off |
|  | Impulse concentric asymmetry | Asymmetry was calculated as: (involved – uninvolved) / (involved + uninvolved) x 100 and (left – right) / (left + right) x 100 for ACLR and healthy controls, respectively |
|  | Impulse eccentric deceleration | Eccentric phase, the time from initiation of the jump to zero center of mass velocity. The eccentric deceleration phase was defined as the time interval from the maximum negative velocity to zero velocity. Eccentric deceleration was calculated via time integration of the force-time curve during the eccentric deceleration phase |
|  | Impulse eccentric deceleration asymmetry | Asymmetry was calculated as: (involved – uninvolved) / (involved + uninvolved) x 100 and (left – right) / (left + right) x 100 for ACLR and healthy controls, respectively |
|  | Concentric peak vGRF | Not normalized to body weight (based on the values) |
|  | Concentric Peak vGRF asymmetry | Asymmetry was calculated as: (involved – uninvolved) / (involved + uninvolved) x 100 and (left – right) / (left + right) x 100 for ACLR and healthy controls, respectively |
|  | Eccentric deceleration RFD | Not operationalized |
|  | Eccentric deceleration RFD asymmetry | Asymmetry was calculated as: (involved – uninvolved) / (involved + uninvolved) x 100 and (left – right) / (left + right) x 100 for ACLR and healthy controls, respectively |
|  | Eccentric mean GRF | Not normalized to body weight (based on the values) |
|  | Eccentric mean GRF asymmetry | Asymmetry was calculated as: (involved – uninvolved) / (involved + uninvolved) x 100 and (left – right) / (left + right) x 100 for ACLR and healthy controls, respectively |
|  | Peak vGRF (Landing) | Not normalized to body weight (based on the values) |
|  | Peak vGRF (Landing) asymmetry | Asymmetry was calculated as: (involved – uninvolved) / (involved + uninvolved) x 100 and (left – right) / (left + right) x 100 for ACLR and healthy controls, respectively |
| Schmitt 2015 | Eccentric peak vGRF_(normalized)_ | During the landing phase. Forces were normalized to body weight. |
|  | Eccentric loading rate_(normalized)_ | Peak vGRF divided by the time to reach peak; BW/seconds |
| Shimizu 2019 | Peak vGRF_(normalized)_ | All data were analyzed during the landing phase of the task (stance phase). The stance phase of the task was defined as initial contact to toe off (vGRF > 20 N) and was time normalized to 101 points. Forces were normalized to body weight. |
| Shimizu 2020 | Eccentric peak vGRF_(normalized)_ | The stance phase of the task was defined as initial contact to toe-off and was time normalized to 101 points. All data were analyzed during the landing phase of the task (stance phase). The peak vGRF was calculated during the (first 50% of stance phase). Forces were normalized to body weight. |
| Shimizu 2019a | Eccentric peak vGRF_(normalized)_ | The stance phase of the task was defined as initial contact to toe off and was time normalized to 101 points. All data were analyzed during the landing phase of the task (stance phase). The peak vGRF was calculated during the (first 50% of stance phase). Forces were normalized to body weight. |
|  | vGRF Impulse | The vGRF impulse during the stance phase of the drop-jump task was calculated as the time-based integral of the vGRF |
| vGRF: vertical ground reaction force; ACLR: anterior cruciate ligament reconstruction; GRF: ground reaction force; CMJ: countermovement jump; hGRF: horizontal ground reaction force; CoP: center of pressure; TTS: time to stabilization; SD: standard deviation; BCM: body center of mass; m/s: meter per second; IC: initial contact; g: gravity; N: newton; RSI: reactive strength index; RSR: reactive strength ratio; S: second; DJ: drop jump; mlGRF: medial lateral ground reaction force; CoM: center of mass; AP: anterior posterior; ML: medial lateral; RFD: rate of force development; BW: body weight | | |

**Appendix IV – Non-discriminatory parameters during double-leg drop jumps**
